# Autophagy is an upstream mediator of chromatin dynamics in normal and autoimmune germinal center B cells

**DOI:** 10.1172/JCI178920

**Published:** 2025-05-15

**Authors:** Marta C. Sallan, Filip Filipsky, Christina H. Shi, Elena Pontarini, Manuela Terranova-Barberio, Gordon Beattie, Andrew Clear, Michele Bombardieri, Kevin Y. Yip, Dinis Pedro Calado, Mark S. Cragg, Sonya James, Mathew Carter, Jessica Okosun, John G. Gribben, Tanya Klymenko, Andrejs Braun

**Affiliations:** 1 Barts Cancer Institute, Queen Mary University of London, Charterhouse Square, London, United Kingdom.; 2 Sanford Burnham Prebys Medical Discovery Institute, La Jolla, California, USA.; 3 Centre of Experimental Medicine and Rheumatology, William Harvey Research Institute, Charterhouse Square, London, United Kingdom.; 4 CRUK City of London Centre Single Cell Genomics Facility and; 5 Genomics Translational Technology Platform, UCL Cancer Institute, University College London, London, United Kingdom.; 6 Immunity and Cancer Laboratory, Francis Crick Institute, London, United Kingdom.; 7 Centre for Cancer Immunology, Faculty of Medicine, University of Southampton, Southampton, United Kingdom.; 8 School of Biological and Behavioural Sciences, Queen Mary University of London, London, United Kingdom.

**Keywords:** Autoimmunity, Cell biology, Immunology, Autoimmune diseases, Autophagy

## Abstract

Germinal center (GC) B cells are pivotal in establishing a robust humoral immune response and long-term serological immunity while maintaining antibody self-tolerance. GC B cells rely on autophagy for antigen presentation and homeostatic maintenance. However, these functions, primarily associated with the light zone, cannot explain the spatiotemporal autophagy upregulation in the dark zone of GCs. Here, combining imaging, molecular, and genomic approaches, we defined a functional mechanism controlling chromatin accessibility in GC B cells during their dark zone transition. This mechanism links autophagy and nuclear lamin B1 dynamics with their downstream effects, including somatic hypermutation and antibody affinity maturation. Moreover, the autophagy–lamin B1 axis is highly active in the aberrant ectopic GCs in the salivary glands of Sjögren’s disease, defining its role in autoimmunity.

## Abstract 2

In this study, we find autophagy is spatially enhanced in the dark zone of canonical germinal centres and proliferating B cells in GC-like structures in Sjogren’s disease controlling Lamin B1 nuclear abundance. The autophagy-Lamin B1 axis is key to regulating chromatin accessibility.

## Graphical abstract

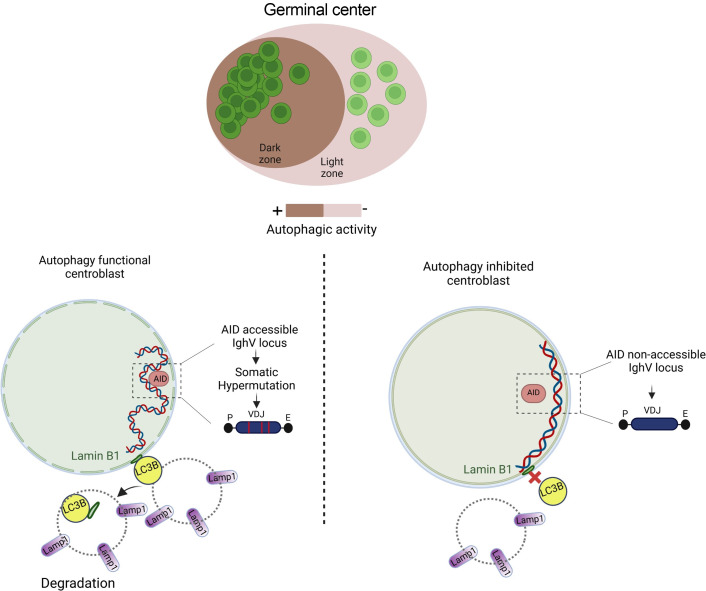

## Introduction

Macroautophagy (hereafter, autophagy) is a cellular mechanism that involves the degradation and recycling of cellular components. It plays a crucial role in a broad spectrum of cellular and organismal functions, including innate and adaptive immune responses (1). In this context, autophagy is crucial for the healthy self-renewing population of lymphoid precursors and for maintaining immunological memory (2–5).

Although initial studies indicated that autophagy is not essential for the survival of mature B cells that transit germinal centers (GCs) (2, 5, 6), these cells exhibit some of the highest rates of autophagy seen in differentiated B cells (7). Furthermore, canonical and noncanonical autophagy can facilitate B cell receptor (BCR) polarization and B cell antigen internalization, thus supporting B cell differentiation (6, 7). However, these functions do not explain the presence of autophagy in the dark zones (DZs) of lymphoid GCs, including its precise role and significance in the centroblast (CB) population.

During GC reaction, B cells undergo a massive yet rigorous rearrangement of genome architecture. In CBs, the genome architecture adapts to facilitate somatic mutagenesis in chromatin regions containing immunoglobulin variable (IgV) genes (8–10). *IgV* locus, in turn, is functionally and topologically linked to nuclear lamina, and is an integral component of lamina-associated chromatin domains (11), crucial for deaminase-mediated *IgV* mutagenesis in vitro (10). However, the mechanisms governing the regulation of lamin B1 nuclear dynamics in GC B cells remain unknown. Likewise, little is known about the downstream impact of nuclear autophagy on DZ GC dynamics in normal and pathological conditions, as well as on the production of high-affinity immunoglobulins during adaptive immune response.

One such potential mechanism, linking autophagy to the downstream lamina-associated B cell genome dynamics, can be extrapolated from prior studies that reported lamin B1 as a specific substrate for autophagic degradation in RAS-activated fibroblasts (12, 13). These and other observations, anecdotally linking nuclear autophagy to neurodegeneration (14), differentiation (15), senescence (16), and cancer (17), suggest the existence of an epigenetic functional axis linking upstream autophagy with downstream broad-scale chromatin changes and somatic mutagenesis.

In ectopic GC-like structures in autoimmunity, autophagy synergizes with autoimmune checkpoint subversion in self-reactive B cells, leading to their aberrant activation and downstream BCR signaling (18, 19). Likewise, maladaptive autophagy is a metabolic hallmark in the pathogenesis of Sjögren’s disease (SD) (20), which correlates with its histological severity (21). Interestingly, increased autophagic activity in SD is restricted to ectopic GC-like structures and is not detectable in circulating SD lymphocytes (21), suggesting shared mechanisms of autophagy regulation in normal and autoimmune GC B cells. Currently, there is no functional explanation for autophagy’s role in the molecular pathogenesis of SD and other autoimmune diseases. In this study, we aimed to define the functional mechanisms explaining why autophagy is upregulated in the DZ of GC and to explore its role in autoimmunity.

## Results

### GC B cells upregulate selective autophagy in the DZ area.

**Figure 1:**
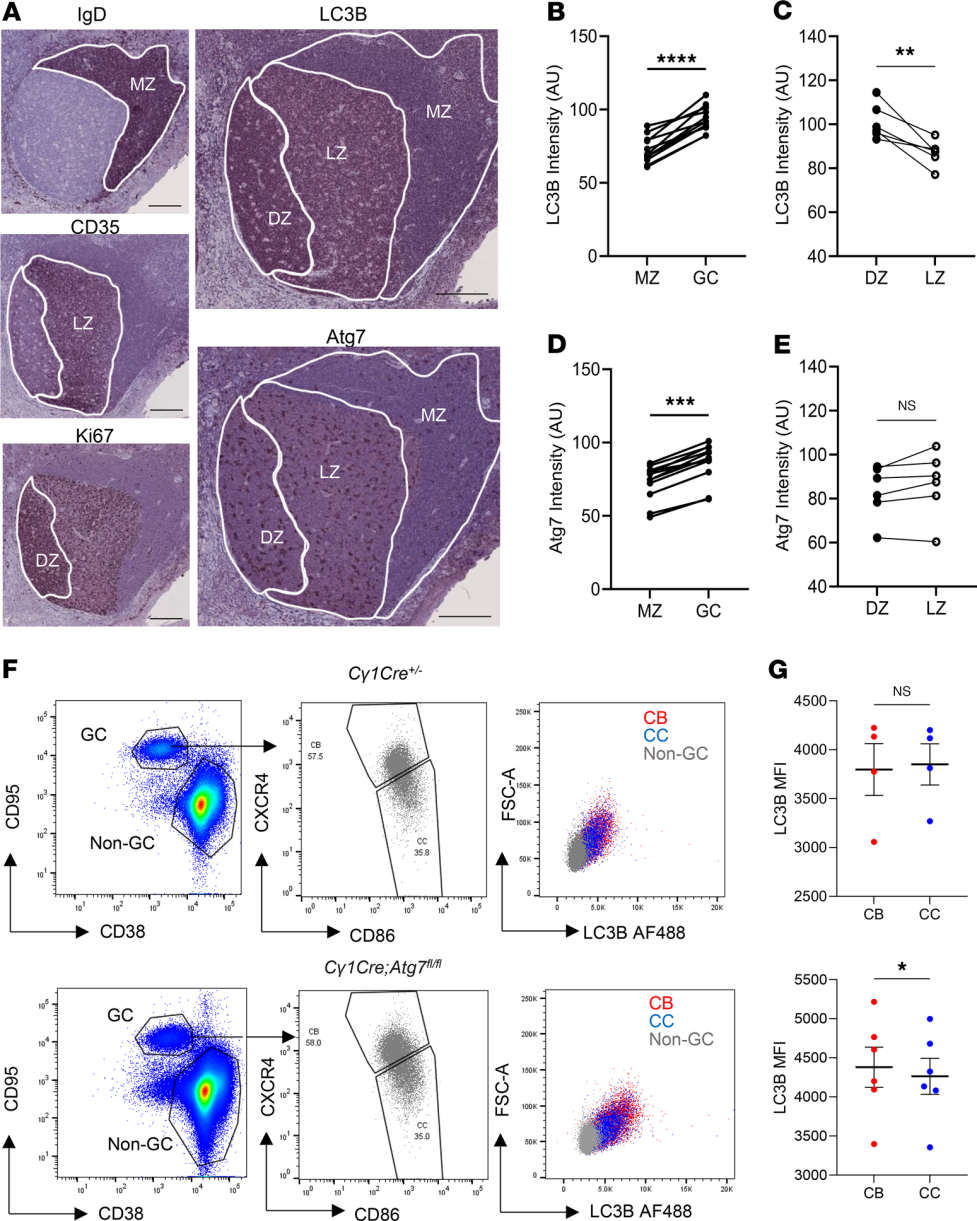
GC B cells upregulate autophagy in the DZ area. (**A**) Representative immunohistochemistry images on sequential slides of human GC from a reactive tonsil. Tonsils were stained with anti-IgD, anti-CD35, anti-Ki67, anti-LC3B, and anti-Atg7 antibodies, and nuclei were counterstained with hematoxylin (blue). Three to 6 GCs per tonsil were analyzed from 3 patients. Scale bars: 200 μm. (**B**) LC3B intensity was compared in MZ (IgD^+^CD35^–^Ki67^–^) and GC (IgD^–^CD35^+^Ki67^+^). Statistics were obtained using paired 2-tailed Student’s *t* test. (**C**) LC3B intensity was compared in DZ (IgD^–^Ki67^+^CD35^–^) and LZ (IgD^–^Ki67^–^CD35^+^) areas. Statistics were obtained using paired 2-tailed Student’s *t* test. (**D**) Atg7 intensity was compared in MZ (IgD^+^CD35^–^Ki67^–^) and GC (IgD^–^CD35^+^Ki67^+^). Statistics were obtained using paired 2-tailed Student’s *t* test. (**E**) Atg7 intensity was compared in DZ (IgD^–^Ki67^+^CD35^–^) and LZ (IgD^–^Ki67^–^CD35^+^) areas. Statistics were obtained using paired 2-tailed Student’s *t* test. (**F**) CB (VD^–^B220^+^CD38^–^CD95^+^CXCR4^+^CD86^–^) and CC (VD^–^B220^+^CD38^–^CD95^+^CXCR4^–^CD86^+^) gated populations followed by LC3B-overlaid dot plots in control mice (WT *Atg7* *Cγ1Cre^+/–^*) and autophagy-impaired mice (*Cγ1Cre^+/–^* *Atg7^fl/fl^*). (**G**) Plots showing LC3B median fluorescence intensity (MFI) at 7 dpi in 2 independent experiments. Statistical analysis was performed using paired 2-tailed Student’s *t* test. Statistical significance: *P* < 0.05 (*), *P* < 0.01 (**), *P* < 0.001 (***), *P* < 0.0001 (****).

To investigate the regional autophagic boost in the GC areas, we first evaluated the distribution of key autophagic machinery components (Figure 1A and Supplemental Figure 1A; supplemental material available online with this article; https://doi.org/10.1172/JCI178920DS1) within human reactive lymphoid follicles (Supplemental Table 1). LC3B (22) was topologically linked to GCs (Figure 1B and Supplemental Figure 1B), more specifically to the DZ, in human reactive tonsils (Figure 1C) and lymph nodes (Supplemental Figure 1C). We also evaluated Atg7 expression in the GC structures and observed the increased Atg7 expression in GC compared with mantle zone (MZ) areas (Figure 1D and Supplemental Figure 1D). A comparable amount of Atg7 was observed in DZ and light zone (LZ) areas (Figure 1E and Supplemental Figure 1E), suggesting upstream negative autophagy regulation in the LZ area. Moreover, multiplex immunohistochemistry on mouse GCs revealed that Atg7 expression was upregulated in mouse GCs compared with MZ B cells (Supplemental Figure 1F). To further investigate the autophagy role in vivo, we generated a GC-specific mouse model in which we inhibited autophagy by using the *Cγ1-cre* recombinase, implementing a controlled *Atg7* knockdown through floxed alleles (Supplemental Figure 2A). Using this model, we further verified LC3B accumulation in CBs and centrocytes (CCs) upon *Atg7* knockout (Supplemental Figure 2B), which was asymmetrically higher in CBs (Figure 1F).

We then used human and mouse RNA-Seq datasets extracted from Victora et al. (23) to perform gene set enrichment analysis for autophagy (AP) molecular signatures (Supplemental Figure 2, C and D). “AP” and “selective AP” were significantly upregulated in the DZ. Complementary to this, “AP regulation” and “negative AP regulation” signatures were significantly upregulated in the LZ, indicating that the autophagy boost in the DZ is actively downregulated in the LZ. Our data suggest that the autophagy pathway, more specifically selective autophagy, is upregulated in the DZ, having differential roles in the DZ and LZ during GC responses.

Next, the in vitro BCR stimulation of BL2 human cells (24) and primary human B cells, in combination with the autophagy blocker hydroxychloroquine (Supplemental Figure 3A), showed an increase of LC3B (Supplemental Figure 3, B and C) and p62 (Supplemental Figure 3, D and E) content as soon as 3.5 hours after activation. These results imply that the upregulation of autophagy in B cells is rapid and specific to BCR signaling. To further verify the specificity of autophagy driven by BCR engagement, we stimulated BCR receptor in BL2 cells in combination with hydroxychloroquine or the BCR inhibitor ibrutinib. Our findings showed that autophagy activity decreased in BCR-engaged cells when treated with ibrutinib (Supplemental Figure 4, A and B). These results support our hypothesis that BCR signaling activates autophagy.

**Figure 2:**
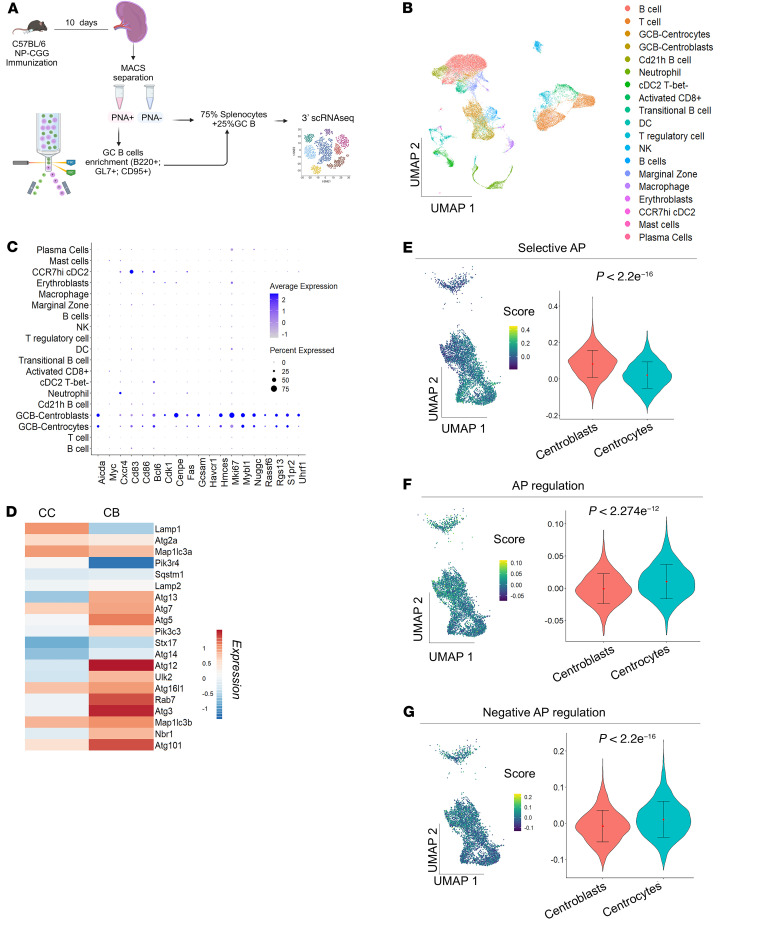
scRNA-Seq of mouse splenocytes reveals enhanced selective autophagy in the centroblast population. (**A**) Schematic experimental setup for the single-cell RNA-Seq approach performed on our control mice (WT *Atg7* *Cγ1Cre^+/–^*). (**B**) UMAP plot showing splenic cell populations identified after 3′ scRNA-Seq. (**C**) Dot plot of GC signature obtained from Glaros et al., 2021 (25). (**D**) Heatmap plotting average expression of 20 canonical autophagy markers comparing CCs and CBs. (**E**–**G**) AddModuleScore analysis visualization and violin plot showing differences in signature expression. Welch’s 2-sample *t* test was performed. “Selective autophagy” (**E**), “autophagy regulation” (**F**), and “negative autophagy regulation” (**G**) gene sets were analyzed.

To dissect the spatial impact of autophagy on GCs, we generated an in vivo immune response and performed single-cell RNA-Seq (scRNA-Seq) analysis in splenocytes from control *Cγ1Cre^+/–^* mice at the peak of GC reaction (Figure 2A). The corresponding uniform manifold approximation and projection (UMAP) plot revealed the presence of all main spleen cell populations (Figure 2B). Gene expression signatures, in turn, showed that the GC population was represented in cluster 2 (GCB-centrocytes [LZ]) and cluster 4 (GCB-centroblasts [DZ]) (23, 25, 26) (Figure 2C).

We then explored the expression of 20 canonical autophagy markers in the CCs and CBs and found that most of these markers were upregulated in the CB population (Figure 2D).

Further focusing on the spatial autophagy regulation in our system, we next visualized single-cell scores for the activity of predefined autophagy gene sets. The “selective AP” signature was upregulated in the CB cluster (Figure 2E), further verifying an autophagy cargo-selective role in the DZ. The “AP regulation” and “negative AP regulation” signatures, in turn, were all confined to CCs (Figure 2, F and G), indicating that autophagy is transcriptionally downregulated in the LZ.

Using the mouse gene set Biocarta_BCR_Pathway, we then interrogated our scRNA-Seq data for upregulated genes involved in the BCR cascade in DZ-associated B cells. We found significant transcriptional upregulation of 8 of the 31 genes in CBs (Supplemental Figure 4C). Subsequently, Ingenuity Pathway Analysis (IPA) mapped the upregulated BCR genes to the autophagy pathway. PPP3CB/calcineurin emerged as the most direct potential mediator, activating transcription factor EB (TFEB), which drives autophagy activation (Supplemental Figure 4D).

Our results demonstrate that autophagy is functionally upregulated in the human and mouse DZ areas, defining a cargo-specific autophagy role during the GC reaction initiated by BCR stimulation.

### Autophagy controls nuclear lamin B1 content in GC B cells.

To investigate whether there is a link between the cargo-specific autophagy pathway in DZ-associated CBs and lamin B1 dynamics, we first performed confocal analysis of the subcellular colocalization between the nuclear lamina and autophagic machinery in vitro in control and BCR–cross-linked (BCRx) BL2 cells (Supplemental Figure 5A). The BCR-engaged cells showed a substantial structural overlap between lamin B1 and LC3B (Supplemental Figure 5, B and D) or lysosomal marker LAMP1 (Supplemental Figure 5, C and D). This overlap was 1.5- to 5-fold more frequent in BCRx cells than in control, with much of this interaction occurring at the nuclear periphery.

**Figure 3:**
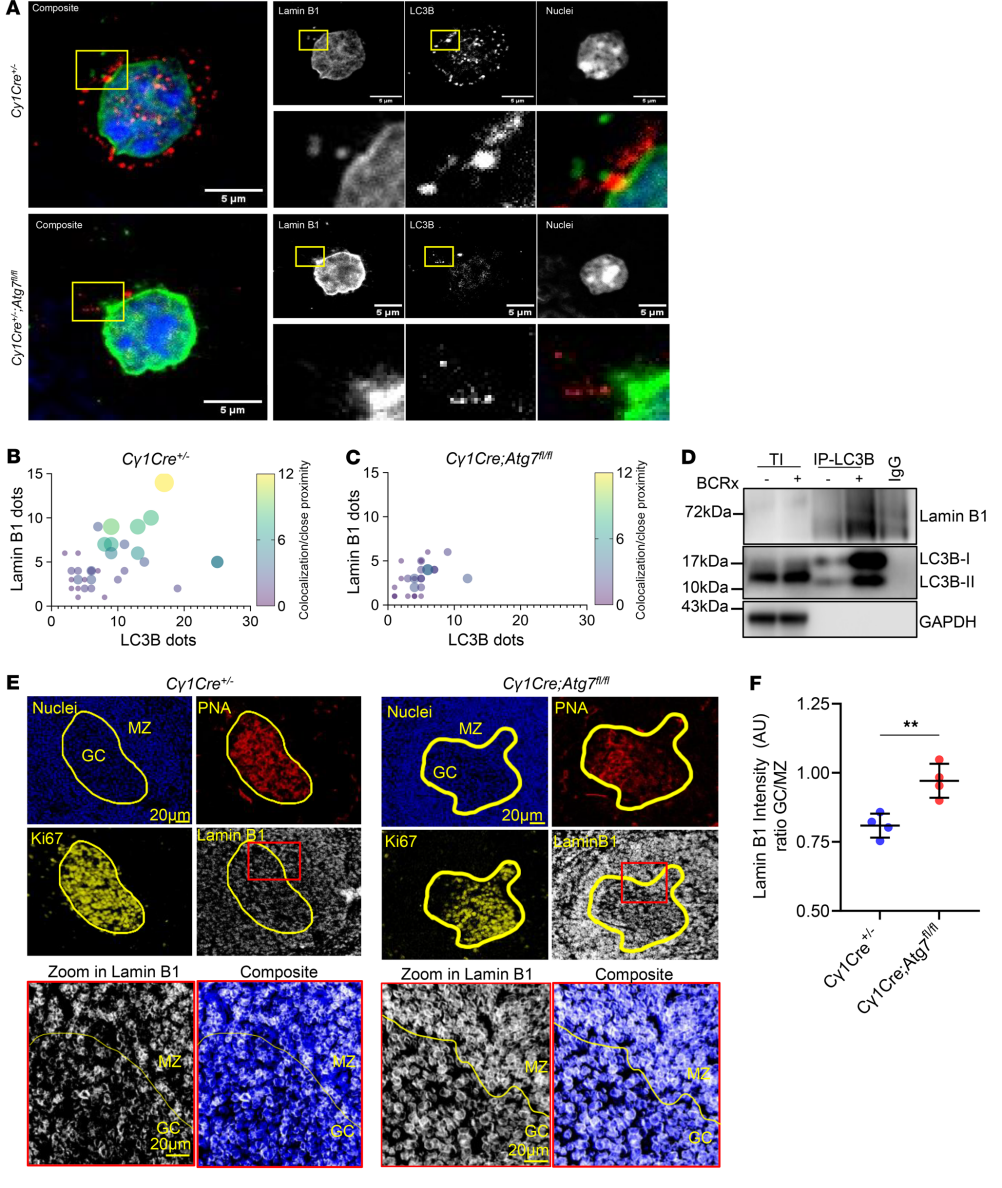
Autophagy directly interacts with lamin B1, controlling its expression, in GCs. (**A**) Lamin B1 and LC3B representative images from MACS-separated PNA^+^ cells from 10 dpi. PNA^+^ cells were stained with anti-LC3 (red) and anti–lamin B1 (green) antibodies and analyzed with a ZEISS LSM 880 oil 63× confocal microscope with 4.0 zoom. Scale bars: 5 μm. (**B** and **C**) Bubble chart showing relationship between LC3B-positive (*x* axis) and lamin B1–positive (*y* axis) dots per cell in PNA^+^ *Cγ1Cre^+/–^* and *Cγ1Cre Atg7^fl/fl^* cells. Bubble size and color indicate colocalization. *n* = 2 per mouse genotype. (**D**) LC3 immunoprecipitation of BL2 cells upon BCR activation. (**E**) Representative multiplex immunohistochemistry images of *Cγ1Cre^+/–^* and *Cγ1Cre Atg7^fl/fl^* GCs, 10 dpi with NP-CGG. Spleen cuts were stained with anti-PNA (red), anti-Ki67 (yellow), and anti–lamin B1 (gray). Nuclei were counterstained with hematoxylin (blue). Scale bars: 20 μm. (**F**) Lamin B1 intensity (AU) ratio between GC and MZ measured in at least 3 follicles per mouse in *n* = 4 mice from 2 independent experiments. Horizontal bars represent the mean ± SEM. Statistics were obtained using 2-tailed unpaired Student’s *t* test. Statistical significance: *P* < 0.01 (**).

To validate the interaction between autophagy machinery and lamin B1 in GC B cells in vivo, we first obtained peanut agglutinin (PNA) isolated GC B cell fractions 10 days post-immunization (dpi) (Figure 3A). Our results show decreased lamin B1 and LC3B cytoplasmic foci in *Cγ1Cre* *Atg7^fl/fl^* PNA^+^ B cells coinciding with a significant drop in the paranuclear lamin B1–LC3B interaction (Figure 3, B and C). These results verify that during GC reaction, LC3B colocalizes with lamin B1 at the nuclear periphery and show that autophagy controls lamin B1 nuclear abundance in physiological in vivo conditions. To further validate the biochemical autophagy-lamina interaction in our system, we performed an LC3B immunoprecipitation. Figure 3D demonstrates that lamin B1 and LC3B interaction increased upon BCR engagement, thus validating that BCR activation leads to direct interaction between lamin B1 and the autophagy protein LC3B.

We next investigated the in vivo effect of autophagy on GC lamin B1 turnover. We used multiplex immunohistochemistry quantification to assess the impact of *Atg7* loss on lamin B1 nuclear incorporation dynamics in mouse GC B cells. The GC-specific *Atg7* deletion in *Cγ1Cre* *Atg7^fl/fl^* mice (Supplemental Figure 5, E–G) coincided with constant lamin B1 levels in the GC at 10 dpi (Figure 3E, bottom) as opposed to control *Cγ1Cre*^+/–^ mice, in which nuclear lamin B1 was reduced (Figure 3, E and F). Quantitative PCR analysis of *LMNB1* verified that lamin B1 was not modulated at the transcriptional level (Supplemental Figure 6, A and B), implying that autophagy inhibition does not reshape *LMNB1* transcript abundance in autophagy-inhibited GC B cells, and verifying that the lamin B1 nuclear fluctuation in GCs is a consequence of posttranslational events that include autophagy regulation.

We further translated these observations to human reactive tonsils and lymph nodes. We performed multiplex Ki67 and lamin B1 IHC staining using the same human samples we previously stained for LC3B and Atg7 markers (Supplemental Figure 6C). We observed that lamin B1 was reduced in the GC compared with the MZ area from human tonsils and reactive lymph nodes (Supplemental Figure 6, D and E), as previously described in the mouse GC setting (10), suggesting a cross-species role of the autophagy–lamin B1 axis in secondary lymphoid tissues.

To functionally validate the association between autophagy and lamin B1, we first inhibited autophagic flux in vitro using *Atg7* RNA interference (27), simultaneously with BCRx (Supplemental Figure 6F). Immunofluorescence-based quantification revealed that lamin B1 nuclear levels remained stable in autophagy-inhibited control samples. In contrast, BCRx cells showed significantly reduced nuclear lamin B1 3.5 hours after induction. However, the observed lamin B1 reduction was reversed by cotreatment of BCRx cells with *Atg7* RNA interference (Supplemental Figure 6, G and H).

Taken together, our data demonstrate that LC3B interacts with the nuclear protein lamin B1, which can be regulated through the canonical autophagy pathway.

### Autophagy is involved in regulating the chromatin landscape.

One of the main features of GC B cells during an adaptive immune response is their capacity to reorganize their genome, allowing transcription of cell type–specific gene networks (28, 29). We next hypothesized that the autophagy–lamin B1 functional link might play a prominent role in regulating the downstream genomic landscape in B cells, including chromatin accessibility for somatic mutagenesis and transcriptional profiling.

**Figure 4:**
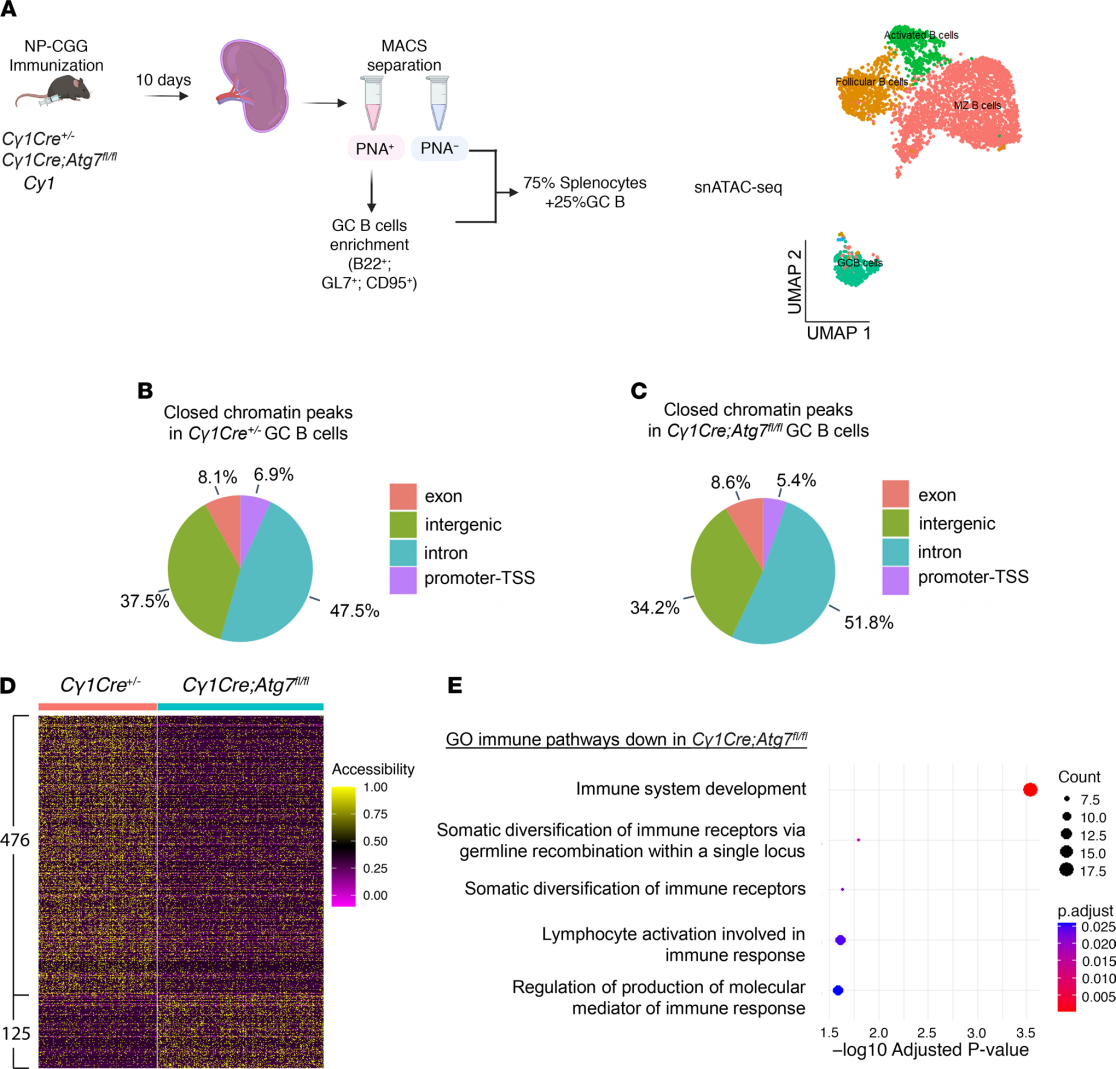
Autophagy regulates GC B cell chromatin landscape. (**A**) Schematic visualization of the snATAC-Seq experiment and B cell subset using UMAP representation. (**B** and **C**) Pie charts detailing the distribution of variable closed chromatin peaks (Exon, Intergenic, Intron, and Promoter-TSS) with a fold change ≥ 10 found in the GC B cell cluster in *Cγ1Cre^+/–^* (**B**) and *Cγ1Cre Atg7^fl/fl^* mice (**C**). (**D**) Heatmap showing differentially accessible chromatin regions in GC cluster (fold change > 1.2, FDR < 0.1, and *P* < 0.05). (**E**) Non-redundant biological terms for DA genes obtained in **D** using clusterProfiler for gene ontology (GO) enrichment analysis. Only GO terms related to immunity are shown; adjusted *P* value < 0.05.

We performed single-nucleus assay for transposase-accessible chromatin using sequencing (snATAC-Seq) on splenocytes isolated from *Cγ1Cre^+/–^* and *Cγ1Cre Atg7^fl/fl^* animals at 10 dpi (Figure 4A and Supplemental Figure 7A), identifying the GC B cell population using established markers (25) (Supplemental Figure 7B). The *Atg7* loss did not produce any changes in cluster proportions at 10 dpi (Supplemental Figure 7C), highlighting that any downstream functional changes observed would not be the result of alterations in steady-state proportions of cells.

The analysis of differentially closed chromatin variable peaks (fold change > 10) in *Cγ1Cre^+/–^* (Figure 4B) and *Cγ1Cre Atg7^fl/fl^* mice (Figure 4C) revealed that a significant percentage of exons and introns were indeed sensitive to *Atg7* loss in comparison with the littermate controls. Both *Cγ1Cre^+/–^* and *Cγ1Cre* *Atg7^fl/fl^* mice shared a high percentage of common open chromatin peaks in the exon and promoter regions, as expected from a GC B cell (Supplemental Figure 7D) and common closed chromatin peaks (Supplemental Figure 7E).

The analysis of differentially accessible (DA) peaks revealed a 3.8-fold decrease in chromatin accessibility in the GC cluster of *Cγ1Cre Atg7^fl/fl^* cells (fold change > 1.2, FDR < 0.1) (Figure 4D), validating the involvement of autophagy in chromatin relaxation, potentially through lamin B1 regulation. We next mapped gene body regions that overlapped DA peaks in *Cγ1Cre^+/–^* and *Cγ1Cre Atg7^fl/fl^* cells from the GC B cell cluster (Supplemental Table 2). We found that 411/601 DA peaks in Figure 4D overlapped with the gene body regions of 410 genes. Among these 410 genes, 379 overlapped with 1 DA peak per each gene, whereas 27 genes overlapped with 2, and 4 genes overlapped with 3 DA peaks. To associate the DA body genes with potential transcriptional perturbations in groups of coordinately regulated genes, we used clusterProfiler for gene ontology enrichment analysis. We visualized the nonredundant immune system ontologies (adjusted *P* value < 0.05). In autophagy-inhibited phenotype, we identified a significant negative enrichment of 12 Gene Ontology (GO) terms involved in (a) immune system development, (b) B cell signaling, and (c) somatic diversification of immune receptors via germline recombination within a single locus (Figure 4E), suggesting compromised GC B cell development.

### Autophagy controls somatic mutations in GC B cells.

**Figure 5:**
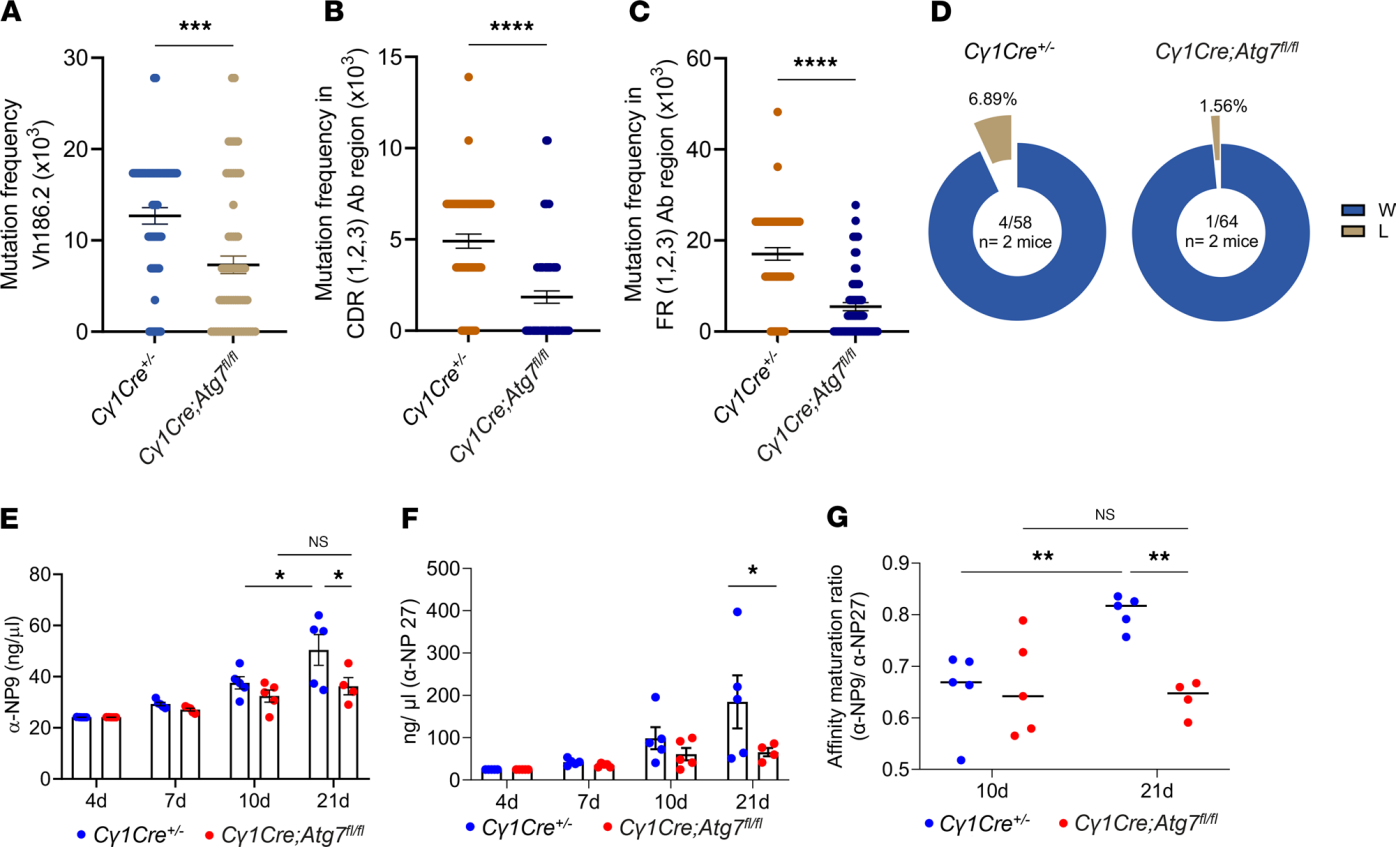
Reduced somatic mutations and antibody affinity in autophagy-impaired GC B cells. (**A**) Mutation frequency in *V_H_186.2 IgV* region from sorted GC B cells. Average ± SEM; *n* = 2 mice in 2 independent experiments. Unpaired 2-tailed Student’s *t* test coupled to Mann-Whitney. (**B** and **C**) Mutation frequency in CDR (**B**) and FR (**C**) in *V_H_186.2 IgV* region from sorted GC B cells of *Cγ1Cre^+/–^* and *Cγ1Cre Atg7^fl/fl^* mice 10 dpi. Unpaired 2-tailed Student’s *t* test coupled to Mann-Whitney. (**D**) Percentage of NP high-affinity clones (carrying the W33L mutation in CDR1) in sorted GC B cells of *Cγ1Cre^+/–^* and *Cγ1Cre Atg7^fl/fl^* mice 10 dpi. Numbers in the center of each pie chart refer to the number of individual sequences analyzed. (**E** and **F**) Titers of IgG1 anti-NP antibodies were detected with NP_9_ (high-affinity) (**E**) or NP_27_ (total) (**F**) probes in a time-course experiment (at least 4 mice per group). Data are presented as mean ± SEM representative of 3 independent experiments. Two-way ANOVA with Šidák’s multiple-comparison test. (**G**) Affinity maturation ratio (anti-NP_9_/anti-NP_27_) over time. Two-way ANOVA with Tukey’s multiple-comparison test. Statistical significance: *P* < 0.05 (*), *P* < 0.01 (**), *P* < 0.001 (***), *P* < 0.0001 (****).

We next performed mutational sequencing analysis of the *IgV_H_186.2* region from sorted B220^+^CD95^+^GL7^+^ GC B cells as previously described (30). Figure 5A demonstrates that *Cγ1Cre* *Atg7^fl/fl^* GC B cells displayed significantly lower overall mutational load than *Cγ1Cre^+/–^* control cells. Specifically, we found that more than a third of the *Cγ1Cre* *Atg7^fl/fl^* *V_H_186.2* clones had no point mutations at all, with a further 45% having only 1–3 mutations. The latter was in stark contrast with *Cγ1Cre^+/–^* control cells, where most clones contained more than 4 mutations (Supplemental Figure 8A). These results suggest that autophagy has an upstream physiological impact on somatic mutagenesis in the *IgV_H_186.2* region of GC B cells.

Next, we tested the distribution of de novo point mutations across hypervariable complementarity-determining regions (CDRs) and structural framework regions (FRs) (Figure 5, B and C). A deep analysis of replacement and silent mutation positions across the V_H_186.2/Jh2 cluster (CDR1, 2, and 3; FR1, 2, and 3) revealed significantly lower nucleotide substitution frequency in *Cγ1Cre* *Atg7^fl/fl^* animals as compared with control (Supplemental Figure 8, B–E). There was no significant impact of *Atg7* loss on the replacement versus silent mutation ratio (Supplemental Figure 8F), suggesting that de novo mutations follow an activation-induced cytidine deaminase–mediated (AID-mediated) stochastic process.

We next analyzed the same *V_H_186.2* region for the presence of a characteristic tryptophan-to-leucine substituting mutation at position 33 (W33L), which defines the encoding of a high-affinity anti-NP BCR when paired with an Igλ1 light chain (31). We observed a 4-fold suppression (6.89% vs. 1.56%) of W33L substitution in *Cγ1Cre* *Atg7^fl/fl^* GC B cells compared with control cells, suggesting a functional impact of autophagy machinery on generation of high-affinity BCRs (Figure 5D). Complementarily, we found that the secretion of high-affinity anti-NP_9_ IgG1 was significantly impaired in *Cγ1Cre* *Atg7^fl/fl^* animals (Figure 5E), with substantially flatter antibody production dynamics (Figure 5F). As a result, the affinity maturation ratio was considerably decreased upon autophagy inhibition (Figure 5G).

Quantification of GC nuclear AID expression did not reveal any measurable differences in content between *Cγ1Cre* *Atg7^fl/fl^* and *Cγ1Cre^+/–^* mice (Supplemental Figure 8, G and H), indicating that, indeed, autophagy is the master regulator of somatic hypermutation (SHM) in our system.

Together, these data demonstrate that autophagy is a regulator of SHM that translates into functional control over high-affinity immunoglobulin production.

### Autophagy controls the GC transcriptional program in vivo, affecting the cell cycle.

Altered chromatin conformation was a paramount feature of autophagy-inhibited GC B cells. Therefore, the impact of autophagy on chromatin accessibility and immunoglobulin domain mutational load led us to examine further histological and cellular features that could mimic any defects in GC dynamics.

**Figure 6:**
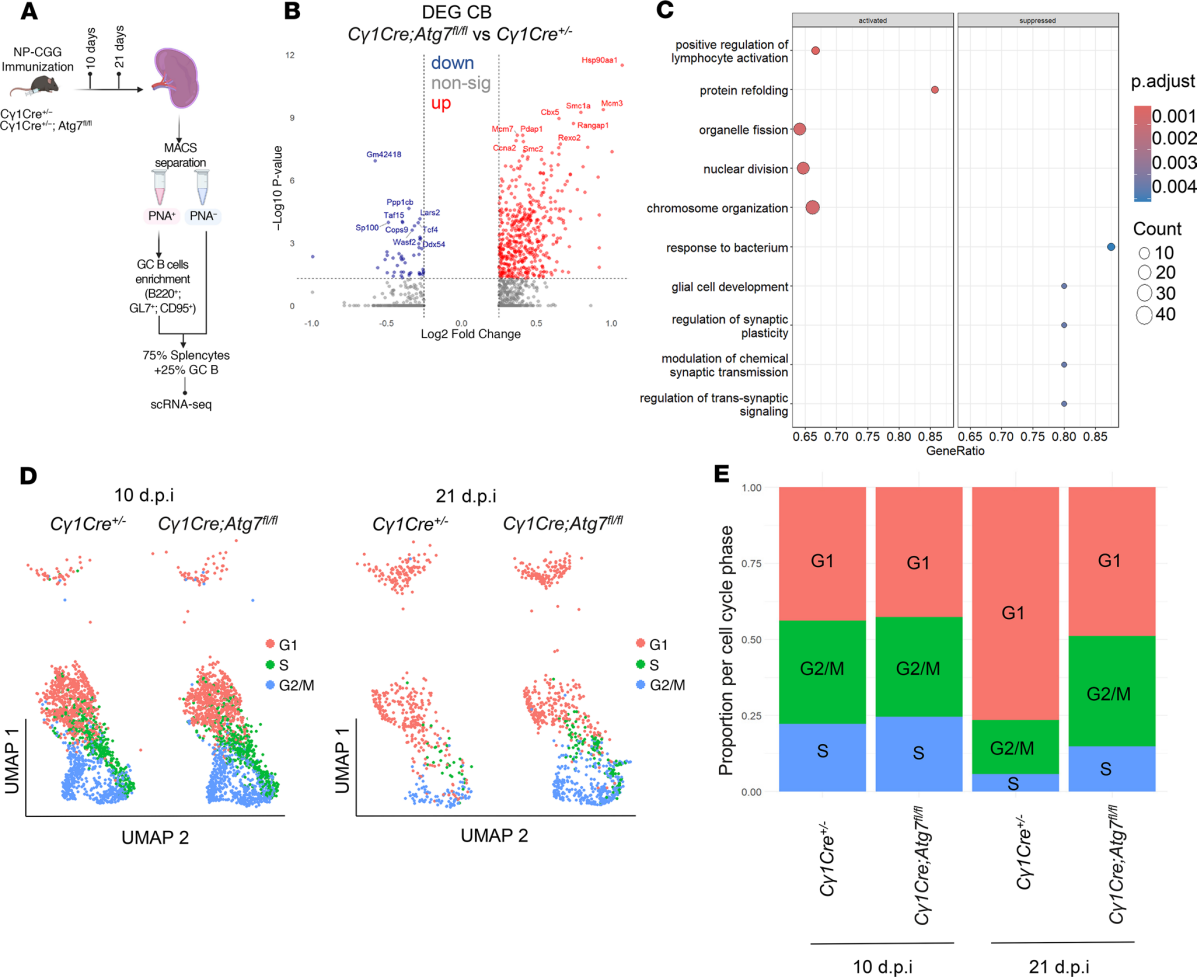
In vivo autophagy orchestrates GC transcriptional program and affects cell cycle. (**A**) Experimental setup for the scRNA-Seq approach performed on *Cγ1Cre^+/–^* and *Cγ1Cre Atg7^fl/fl^* GCs at 10 and 21 dpi. (**B**) Volcano plot showing DEGs in CBs comparing *Cγ1Cre Atg7^fl/fl^* versus *Cγ1Cre^+/–^* at 21 dpi. (**C**) GSEA obtained using the DEGs (adjusted *P* value < 0.05) from **B** plotting the top 10 differentially expressed pathways. (**D**) UMAP plots showing cell cycle in CBs and CC clusters at 10 and 21 dpi. (**E**) Stack plot showing the proportion of cells in G1, G_2_/M, and S phase at 10 and 21 dpi.

To address the effect of *Atg7* loss on gene expression, we performed scRNA-Seq on splenocytes isolated from *Cγ1Cre^+/–^* and *Cγ1Cre Atg7^fl/fl^* mice at 10 dpi (peak of the GC reaction) and 21 dpi (GC finalization) (Figure 6A). We first confirmed that the *Atg7* loss in *Cγ1Cre Atg7^fl/fl^* mice was consistent over time (Supplemental Figure 9, A and B). Despite the *Atg7* knockdown consistency, neither CCs nor CBs showed a significant number of differentially expressed genes (DEGs) and GO pathways affected at the peak of the GC reaction (Supplemental Figure 9, C and D, and Supplemental Tables 3 and 4). In turn, at the GC finalization stage, CBs showed a significant number of DEGs (531 in total, adjusted *P* value < 0.05), indicating the prominent role of autophagy in CB biology (Figure 6B and Supplemental Table 5). In contrast, CCs displayed very few changes when genotypes were compared at 21 dpi (Supplemental Figure 9E and Supplemental Table 5).

Given the transcriptional impact of autophagy in GC finalization, we analyzed DEGs in CBs, revealing cell cycle upregulation in autophagy-deficient GCs. Gene set enrichment analysis (GSEA) also revealed that, among other pathways, *Atg7* loss in CBs was associated with the alterations of the “chromosome organization” pathway (Figure 6C), validating autophagy’s involvement in the assembly and arrangement of chromosomes and related proteins.

As expected, integrated cell cycle analysis of the 2 time points further revealed that the proportion of cells in the G_2_/M and S phases was higher in autophagy-inhibited mice than in *Cγ1Cre^+/–^* controls at 21 dpi (Figure 6, D and E). Therefore, cycle scoring of the single-cell data indicates that *Atg7* loss leads to an accumulation of cells in the G_2_/M and S phases in comparison with control littermates at later time points, while no signs of cell cycle disruption were observed at the peak of the GC reaction. Together, these findings indicate that autophagy plays an essential role in controlling the CB exit from the DZ, ultimately affecting the GC resolution.

### Autophagy is required for DZ to LZ progression.

**Figure 7:**
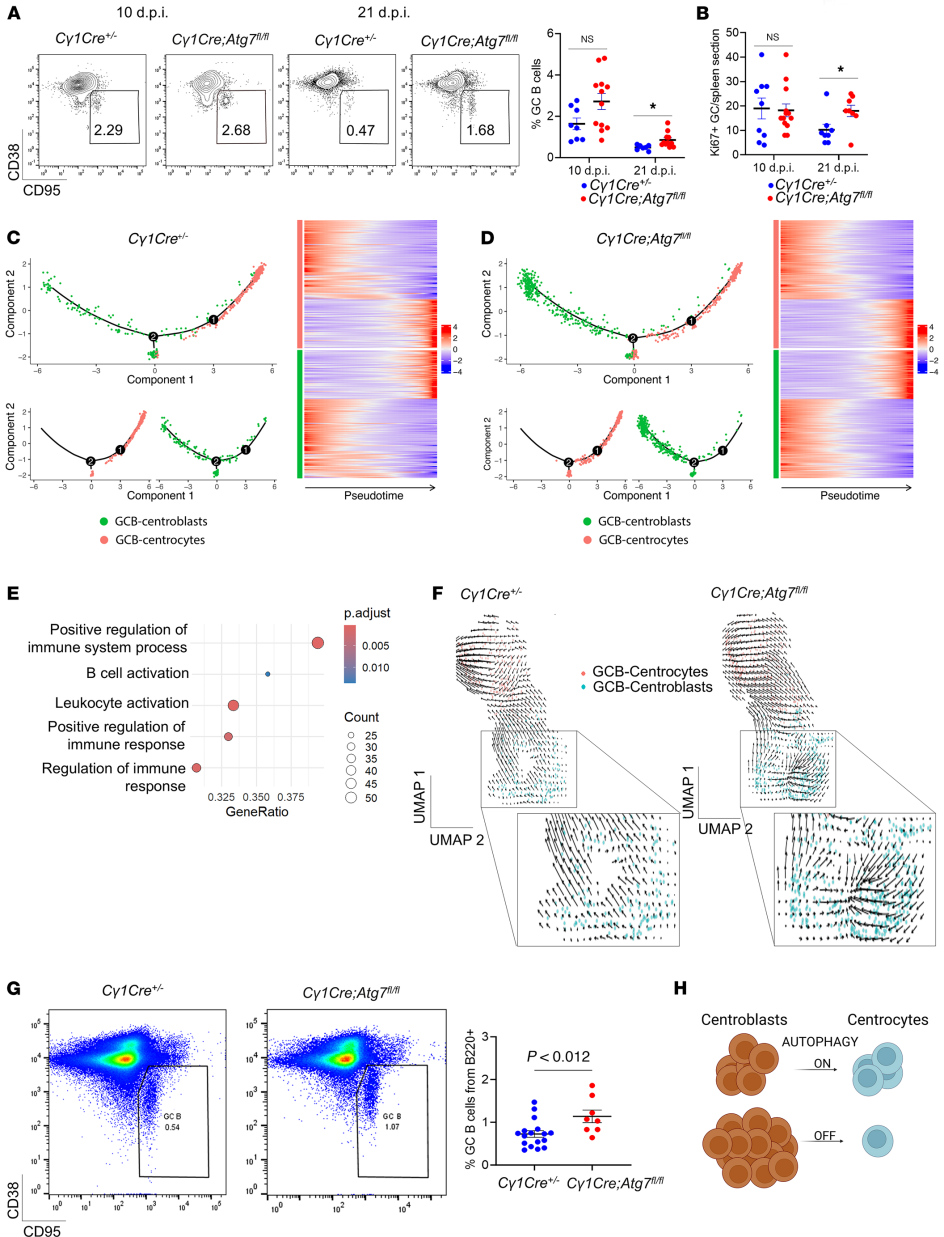
Autophagy is required for DZ to LZ circulation. (**A**) Left: Representative flow cytometry plots from GC B cells (VD^–^B220^+^CD38^–^CD95^+^) at 10 and 21 dpi for the specified genotypes. Right: Summary and quantification of flow cytometry data as shown in the flow plot. Each symbol represents an individual animal from at least 3 independent experiments. Horizontal lines indicate the mean ± SEM. (**B**) GC averages per time point and genotype analyzed. Ki67^+^ GCs were counted, averaged, and compared between genotypes. Horizontal lines indicate the mean ± SEM. Unpaired 2-tailed Student’s *t* test coupled to Mann-Whitney. (**C** and **D**) Visualization of *Cγ1Cre^+/–^* GCB-centroblasts (green) and GCB-centrocytes (red) at 21 dpi. Single-lineage cells were marked with inferred pseudotime by Monocle. Right: Heatmaps displaying changes in gene expression across pseudotime in *Cγ1Cre^+/–^* (**C**) and *Cγ1Cre Atg7^fl/fl^* (**D**). (**E**) GSEA conducted against Gene Ontology biological process (GO-BP) using genes ranked by –log_10_(*P* value) from pseudotime-associated differential expression from **C**. (**F**) RNA velocity analysis UMAP visualization of *Cγ1Cre^+/–^* and *Cγ1Cre Atg7^fl/fl^* cells at 21 dpi. (**G**) Representative flow cytometry plots from GC B cells (VD^–^B220^+^CD38^–^CD95^+^) and quantification at 35 dpi. Data are presented as mean ± SEM representative of *n* = 2 independent experiments with at least 8 animals per genotype. (**H**) Schematic representation of the autophagy inhibition effect of CB to CC transition. Statistical significance: *P* < 0.05 (*).

Given the differences in cell cycle redistribution, we next aimed to define the effect of *Atg7* loss on GC dynamics. We initially compared the percentage of GC B cells generated by 4-hydroxy-3-nitrophenyl-acetyl hapten–chicken γ-globulin (NP-CGG) immunization of *Cγ1Cre^+/–^* and *Cγ1Cre* *Atg7^fl/fl^* cohorts. We found the GC B cell proportion was significantly higher in *Cγ1Cre* *Atg7^fl/fl^* mice compared with the control at the late stage of the GC reaction (21 dpi) (Figure 7A). TUNEL assay revealed nonsignificant differences in apoptotic GC B cells in the *Atg7*-knockout GC B cells at 21 dpi and a significantly reduced apoptotic percentage compared with control at 35 dpi (Supplemental Figure 10, A–C), verifying previous observations that autophagy does not decrease GC B cell survival (2, 3, 5). *Cγ1Cre Atg7^fl/fl^* mice also displayed a significantly higher proportion of GCs per spleen at 21 dpi (Figure 7B). Such GCs are likely functionally active, as the pattern of expression of BCL6 protein, required for the formation and maintenance of GCs (8, 32), was indistinguishable between the 2 genotypes (Supplemental Figure 10, D and E).

When reconstructing the cellular dynamics ordering GC B cells, as a function of pseudotime, we observed them in 5 different states (Supplemental Figure 11A). We defined CB as state 1 with the smallest pseudotime and state 4 (CC) as the final state with the highest pseudotime. States 2, 3, and 5 are transitional states between CBs (state 1) and CCs (state 4) (Supplemental Figure 11B), in accordance with the previously described intermediate states of GC B cells (33). When comparing the cell percentages at different stages, we found a significant accumulation of cells in stage 1 (CB) in the *Cγ1Cre Atg7^fl/fl^* samples (Supplemental Figure 11C), suggesting a blockage of GC cells at the CB stage.

Further trajectory analysis (34) at the peak of the GC reaction (10 dpi) showed that the control and *Cγ1Cre Atg7*^fl/fl^ CB and CC populations had no significant differences among them (Supplemental Figure 11D).

The functional dynamics at 21 dpi, however, indicated that autophagy-inhibited CBs remained stopped in their progression to LZ compared with control (Figure 7, C and D), pointing to a CB deceleration or blockage in the DZ area upon *Atg7* loss. We also found a significant number of genes that change as a function of pseudotime in the CB population in control GCs.

We next performed GSEA of the most significantly changed (*P* < 0.001) genes (Supplemental Table 6). The DEGs upregulated over pseudotime from CB control showed gene expression changes that affect pathways involved in lymphocyte development and activation (Figure 7E). In turn, the DEGs upregulated over pseudotime in CBs from *Cγ1Cre* *Atg7^fl/fl^* mice presented no GSEA intersection with any biological pathway (Supplemental Table 7). To further validate GC dynamics, we performed RNA velocity analysis (35) to reveal the rate and direction of change of the spliced and unspliced CB and CC transcriptome. Consistent with our previous quantitative analysis, day 10 dpi did show minor differences in RNA velocity between genotypes (Supplemental Figure 11E). In contrast, 21 dpi revealed prominent differences in RNA velocity in the CB population (Figure 7F), verifying that the *Atg7* loss favors a CB directional change that translates into deceleration of DZ to LZ transition. To verify the functional outcome of the RNA velocity results, we checked the percentage of GC B cells at 35 dpi. Figure 7G shows that the *Cγ1Cre Atg7^fl/fl^* samples at 35 dpi presented a significantly higher percentage of GC B cells, thus validating the trajectory, RNA velocity, and cell cycle analysis. Overall, these results, schematically represented in Figure 7H, define the involvement of autophagy in DZ-associated functions at the termination stage of the GC reaction.

### Autophagy–lamin B1 axis is active in autoimmune ectopic GC-like structures.

Ectopic GC-like structures are a morphological hallmark of autoimmune diseases, including rheumatoid arthritis, Sjögren’s disease, multiple sclerosis, myasthenia gravis, and systemic lupus erythematosus (36). These structures display several functional similarities to the secondary lymphoid organs during adaptive immune response, albeit supporting aberrant affinity maturation, clonal selection, and differentiation of autoreactive B cells (37). In Sjögren’s disease, 20%–40% of patients develop ectopic GC-like structures (38–40), which are associated with more severe systemic manifestations and evolution to mucosa-associated lymphoid tissue B cell lymphoma (41).

**Figure 8:**
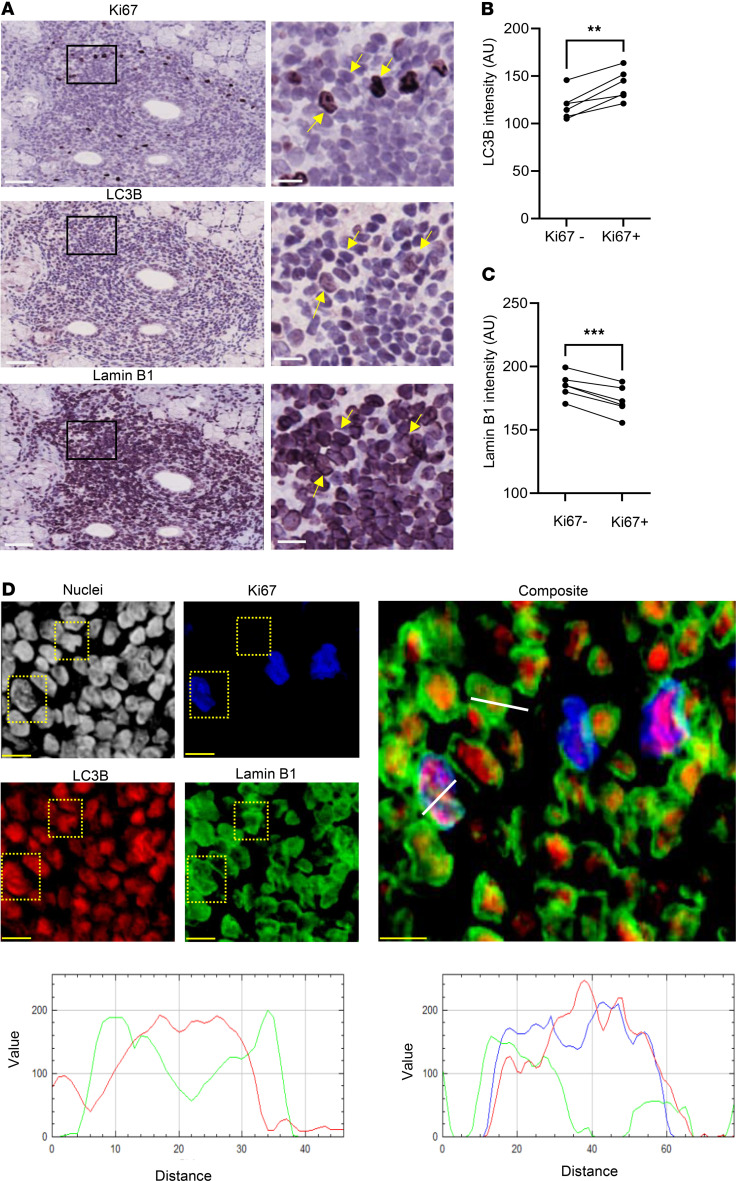
Autophagy–lamin B1 axis is active in autoimmune ectopic lymphoid structures. (**A**) Multiplex immunohistochemistry showing Ki67, LC3B, and lamin B1 staining in the same area in representative SD (case 262); scale bars: 50 μm. Yellow arrows point to the same cells across the multiple staining in zoomed images; scale bars: 10 μm. (**B**) LC3B intensity (AU) in Ki67^+^ versus Ki67^–^ aggregates within the same ectopic lymphoid structure (ELS) per patient. *n* = 6 patients were analyzed. Paired 2-tailed Student’s *t* test. (**C**) Lamin B1 intensity (AU) in ELS with Ki67^+^ aggregates. We compare Ki67^+^ versus Ki67^–^ B cells from the same ELS from the same patient. *n* = 6 patients were analyzed, and 1 ELS per patient was selected. Paired 2-tailed Student’s *t* test. (**D**) Multiplex immunohistochemistry showing nuclei (gray), Ki67 (blue), LC3B (red), and lamin B1 (green) in the same representative SD patient shown in **A**. Scale bars: 10 μm. Plot profile analysis of 3 representative cells. Statistical significance: *P* < 0.01 (**), *P* < 0.001 (***).

Thus, we next tested whether the autophagy–lamin B1 axis is active in the autoimmune ectopic lymphoid structures (Supplemental Table 8). Using CD20 and CD21 as markers, we located areas defined as ectopic GCs (Supplemental Figure 12, A and B) and evaluated LC3B and lamin B1 expression in Ki67^+^ versus Ki67^–^ B cells (Figure 8A and Supplemental Figure 12C).

We observed that Ki67^+^ cells, in the CD21^+^ GC-like area, had higher LC3B levels and reduced lamin B1 nuclear abundance (Figure 8, B–D, and Supplemental Figure 12D), which is consistent with our observations in healthy GCs in human and mouse settings. This pattern suggests that the autophagy–lamin B1 axis is indeed a conserved mechanism throughout GC formation in physiological and pathological conditions.

## Discussion

GC B cells harbor some of the highest levels of autophagy (7), with its suggested association with BCR trafficking and B cell polarization after BCR engagement (7, 42, 43). Here, we find that autophagy is asymmetrically upregulated in the DZ compared with the LZ, and this activation is linked to the selective removal of nuclear lamin B1, which, in turn, is necessary for the accessibility of *IgV* genome loci in CBs. Using multiplex immunohistochemistry in human GCs from reactive tonsils and lymph nodes, complemented with gene dataset analysis, we show that the DZ-associated CBs upregulate autophagy, as compared with CCs. Interestingly, as opposed to LZ cells, CBs upregulate selective autophagy, suggesting that CBs may utilize it to control the abundance of specific subcellular components in an environment. Complementarily, the scRNA-Seq analysis demonstrated the spatial (DZ-associated CB vs. LZ-associated CC) and temporal (GC peak vs. GC resolution) nature of GC regulation by autophagy, correlating with distinct functions of this process in GC expansion and contraction. These data demonstrate a more complex autophagy regulation than initially described, involving spatiotemporal functional components of GC dynamics. LC3B was shown to specifically interact with lamin B1 in RAS-activated fibroblasts as a gateway to induce senescence and as a tumor suppressor mechanism (12, 13). Here, we reveal a complementary LC3B–lamin B1 interplay in a physiological scenario, in which it helps GC B cells to gain chromatin accessibility. At a molecular level, our in vitro and in vivo data show direct LC3B–lamin B1 interaction, further confirming that autophagy is responsible for lamin B1 fluctuations in GC B cells. These results were further validated in human settings. Moreover, the human reactive tonsil and lymph node analysis shows an inverse correlation between elevated LC3B expression and lamin B1 drop, thereby validating the results observed in mouse models and in vitro experiments. The importance of posttranslational autophagic regulation is further confirmed by the fact that lamin B1 mRNA levels remained unaltered in control and *Cγ1Cre Atg7^fl/fl^* mice.

The physiological relevance of autophagic involvement in lamin B1 removal resides in the physical contact that lamin B1 establishes with chromatin (44), translating into one of the primary characteristics of the GC B cells — the capacity to reorganize their genome (28, 29). As a result of this reorganization, immunoglobulin regions become accessible for mutations and BCR substitution (29, 45). In support of this theory, our in vivo snATAC-seq analysis demonstrates that autophagy inhibition results in reduced chromatin accessibility at the peak of the GC reaction, defining its capacity to modulate the genomic architecture of GC B cells.

Affinity maturation is the result of SHM in DZ B cells (46), which is followed by selection by T follicular helper cells in the LZ (47). In this context, our data indicate that the decrease in high-affinity NP antibody production directly results from an impaired SHM due to inefficient lamin B1 nuclear removal in autophagy-deficient GC B cells.

Downstream of SHM regulation, further analysis portrays dynamic temporal changes that affect CB transition to CC, ultimately resulting in CB blockage and delayed GC resolution due to defective canonical autophagy pathway.

Finally, we examined ectopic GC-like structures in SD patients to translate our findings into an autoimmune scenario.

Differently from secondary lymphoid organs, where GCs are easily distinguishable with a visible light and dark zone segregation, aberrant autoimmune ectopic GCs very rarely display recognizable dark and light zones (48). This is due to the absence of the typical anatomical microstructures in ectopic lymphoid tissues, which normally underpin cell migration in physiological conditions.

For that reason, the ectopic GCs require additional markers, such as the long isoform of CD21, to identify the follicular dendritic cell (FDC) networks, which can, in turn, be combined with markers like BCL6. Despite the lack of visual dark/light zone separation, autoimmune ectopic GCs with FDC networks are fully functional (49, 50).

We determined that the lamin B1–LC3B interaction is a common GC restitution mechanism in SD, in which functional ectopic GCs actively participate in activating and differentiating autoreactive B cells. In this context, further studies are warranted to define the relationships between upstream autophagy, chromatin conformational changes, and somatic mutagenesis in SD and other autoimmune conditions.

In summary, this report is, to the best of our knowledge, the first to define a previously uncharacterized epigenetic regulatory mechanism in GC B cells and to explain the presence of selective autophagy in CBs during the GC reaction. Furthermore, it provides what appears to be the first evidence of a cytoplasmic process influencing primary nucleotide substitution and its downstream effects on GC dynamics under both normal and pathological conditions.

## Methods

### Sex as a biological variable

Both male and female participants were included in this study without distinction, and data were analyzed without stratification by sex. The findings are expected to be broadly applicable regardless of sex.

### Antibodies and reagents

The complete list of antibodies and reagents used in this study is available in Supplemental Table 9.

### Cell lines and culture

The BL2 cell line was obtained from the German Collection of Microorganisms and Cell Culture (DSMZ, ACC625). Cells were maintained in RPMI 1640 medium (Thermo Fisher Scientific) supplemented with 10% fetal bovine serum (FBS; Sigma-Aldrich), 1% glutamine (2 mM; Gibco, Invitrogen), and 1% penicillin/streptomycin (Gibco, Invitrogen). Cell lines were regularly tested and verified to be mycoplasma negative using the MycoAlert Detection Kit (Lonza).

### RNA interference experiments

ON-TARGETplus ATG7 siRNA (5 nmol) (Entrez 10533, L-020112-00-000) (Dharmacon/Horizon) and ON-TARGETplus nontargeting pool (D-001810-10) (Dharmacon/Horizon) were used for RNA interference experiments. After electroporation (Lonza Nucleofector 2b), cells were seeded into RPMI 1640 medium, 10% FBS, 1% glutamine, and 1% penicillin/streptomycin at 0.25 × 10^6^ cells/mL. Cell viability was evaluated after 24 and 48 hours and Atg7 expression assessed by Western blot at 48 hours.

### Pharmacological modulation of Bruton’s tyrosine kinase signaling and autophagy

BL2 cells were stimulated using anti-IgM coupled to biotin (ANC-141-030, Caltag Mediasystems) or isotype control (ANC-278-030, Ancell). Simultaneously, the unstimulated and stimulated BL2 cells were treated with the autophagy inhibitor hydroxychloroquine (10 μM), and BCR signaling was interrupted using the Bruton’s tyrosine kinase (BTK) inhibitor ibrutinib (10 μM) for 1 hour. Cells were then processed for Western blot applications.

Western blot bands were quantified using ImageJ (NIH). Levels of BTK, phospho-BTK, LC3B-I, and LC3B-II were calculated after correction to total levels of GAPDH. Four independent experiments were performed, and a 1-way ANOVA test was used for multiple comparisons.

### In vitro induction of IgV somatic mutagenesis

In vitro, somatic hypermutation (SHM) was induced by BCR cross-linking as previously described (24), with minor modifications. Peripheral blood mononuclear cells (PBMCs) were isolated as previously reported (52). Primary human B cells were then purified via negative selection using the B Cell Isolation Kit II (Miltenyi Biotec) and LS columns, according to the manufacturer’s instructions.

For both cell lines and PBMC-derived B cells, 2.5 × 10^6^ cells/mL were incubated in 2 mL serum-free RPMI medium with 4 μg/mL biotinylated anti-IgM (clone UCHB1, Caltag Laboratories), 10 μg/mL anti-CD19 (clone RFB9, in-house), and 10 μg/mL anti-CD21 (clone HB135, in-house) for 20 minutes at 4°C. After washing and resuspension in serum-free RPMI medium, BCR cross-linking was performed by addition of 30 μL streptavidin-coated magnetic beads (Dynabeads M280, Thermo Fisher Scientific) and incubation with agitation at 4°C for 15 minutes. Cells were then resuspended in complete RPMI medium containing 10% FBS at a final density of 0.3 × 10^6^ cells/mL and incubated at 37°C for 210 minutes. For cotreatment experiments, chloroquine diphosphate (Sigma-Aldrich) was added at a final concentration of 10 μM during the incubation.

After treatment, cells were harvested and counted. Cytospin was performed by spinning of 7,000 cells per slide at 30*g* for 5 minutes in 50% FBS-supplemented RPMI medium. Slides were fixed with 4% formaldehyde for 20 minutes at room temperature, washed with PBS containing 0.05% Tween 20, and permeabilized in 0.1% Triton X-100 for 20 minutes. Primary antibodies were applied in PBS with 0.1% BSA for 1 hour, followed by Alexa Fluor 400–conjugated secondary antibodies for 45 minutes. After final washes, slides were mounted with DAPI-containing VECTASHIELD and imaged using a CKX41 fluorescence microscope with a ×100 oil objective and CC12 camera (Olympus).

For Nanoimager visualization, cells were added to flow cytometry tubes and stained as outlined above, except 1 μM DAPI was added alongside secondary antibodies. Excess beads were removed via magnetic separation, and cells were deposited in an 18-well μ-Slide (Ibidi) and imaged using an ONI Nanoimager.

### Ighv somatic mutation analysis

Genomic DNA was prepared from sorted GC B cells (B220^+^, CD95^+^, and GL7^+^) obtained from NP-CGG–immunized *Cγ1Cre Atg7^fl/fl^* or *Cγ1Cre^+/–^* mice on day 10 after immunization. For *V_H_186.2* sequencing, *V_H_186.2-JH2* joints were amplified from genomic DNA by PCR using specific primers for the 5′ end of the *V_H_186.2* gene and the 3′ end of the *JH2* gene as described previously (30). *V_H_186.2-JH2* region was amplified from genomic DNA using Pfu DNA polymerase (Promega) with primers and PCR conditions detailed below. The PCR products were then gel-purified with a QIAquick gel extraction kit (QIAGEN) and cloned with the Zero Blunt TOPO PCR cloning kit (Thermo Fisher Scientific). Plasmid DNA, extracted from individual bacterial colonies, was sequenced in an automated sequencer (ABI3730XL, Thermo Fisher Scientific).

We employed the IMGT/V-QUEST system (https://www.imgt.org/IMGT_vquest/) (51) to exclusively use the productive rearranged sequences and identify specific mutational signatures, including W33L mutations.

### Immunofluorescence

Cells were cytospun onto poly-l-lysine–coated microscope slides, and fixed and permeabilized with 4% paraformaldehyde (PFA; Santa Cruz Biotechnology) or methanol. PFA-fixed cells were permeabilized with 0.1% Triton X-100 (Thermo Fisher Scientific). Slides were washed in TBS/0.05% Tween 20 (Thermo Fisher Scientific) and blocked in TBS/0.1% BSA (Sigma-Aldrich). After incubation with primary and secondary antibodies, slides were washed 3 times in TBS/0.05% Tween and counterstained with 1 μg/mL 4′,6-diamidino-2-phenylindole, dihydrochloride (D1306, Thermo Fisher Scientific). Slides were mounted in ProLong Gold Antifade Reagent (Invitrogen). Imaging was performed using a Nikon Ci-L upright epifluorescence microscope and NIS-Elements software or a ZEISS 810 Laser Scanning Microscope equipped with Zen software (ZEISS). In situ fluorescence intensity was measured within the linear fluorescence range using MetaMorph software equipped with an integrated morphometry analysis module or ImageJ software (NIH). Image processing and quantification were performed according to best-practice guidelines for fluorescence microscopy methods (52).

### Immunoprecipitation

Cells were lysed in IP buffer containing 20 mM Tris, pH 7.5, 137 mM NaCl, 1 mM MgCl_2_, 1 mM CaCl_2_, 1% NP-40, and 10% glycerol supplemented with 1:100 Halt protease and phosphatase inhibitor cocktail (Thermo Fisher Scientific) and benzonase (Novagen) at 12.5 U/mL. The lysates were rotated at 4°C for 30–60 minutes. The supernatant was incubated with antibody-conjugated Dynabeads (Life Technologies) and rotated at 4°C overnight. The immunoprecipitate was washed and collected by magnet 5 times with IP buffer and boiled with NuPAGE loading dye (Thermo Fisher Scientific). Samples were analyzed by Western blotting.

### Western blotting

Whole-cell lysates were prepared in 2× NuPAGE LDS sample buffer (Thermo Fisher Scientific) containing 0.1 M DTT. Samples were then separated using 4%–12% pre-cast NuPAGE Novex gels (Invitrogen) and NuPAGE MES SDS Running Buffer (1×) (Invitrogen/Thermo Fisher Scientific) and transferred onto polyvinylidene difluoride membrane (Thermo Fisher Scientific). Membranes were blocked with 5% BSA in TBS/0.05% Tween 20. After incubation with primary and horseradish peroxidase–conjugated secondary antibodies, proteins were visualized using ECL developer (Thermo Fisher Scientific) and ChemiDoc imaging system (Bio-Rad).

### Histology and multiplex immunohistochemistry

After fixation in 10% buffered formalin, paraffin embedding, and cutting, 3 to 4 μm FFPE sections were used for hematoxylin and eosin and immunohistochemical staining. For immunohistochemical staining, FFPE sections were deparaffinized at 65°C, rehydrated, and incubated in boiling 10 mM pH 6.0 citrate buffer (Vector Laboratories) or 10 mM pH 9.0 Tris-HCl buffer (Vector Laboratories). FFPE sections were then blocked in 2.5% goat serum (Vector Laboratories) and stained with primary and secondary horseradish peroxidase–conjugated antibodies, including isotype controls. Slides were then counterstained with hematoxylin, and the signal was detected by peroxidase chromogen substrate VIP staining (Vector Laboratories). Slides were then mounted in DPX (Sigma-Aldrich) and scanned 24 hours later using a NanoZoomer S60 (Hamamatsu) at ×40 original magnification. Stripping and reprobing were performed by incubation of the slides in xylene for 1 hour, rehydration, antigen retrieval, blocking, and staining for the next round of imaging. Image analysis was performed by combination of ImageJ and QuPath software (53) for image alignment and signal quantification, respectively.

### Image alignment and analysis

Human GCs and MZ areas were identified based on combination of 3 markers and histological features: IgD^–^Ki67^+^CD35^–^ (DZ area), IgD^–^Ki67^–^CD35^+^ (LZ area), and IgD^+^Ki67^–^CD35^–^ (MZ area). Mouse GCs were identified using 2 sets of combined markers and histological features: combination 1, PNA^+^B220^+^; combination 2, CD19^+^AID^+^. Mouse MZ areas were identified using histological features and 2 sets of marker combinations: combination 1, PNA^–^B220^+^; combination 2, CD19^+^AID^–^CD3^–^. Chosen regions of interest (ROIs) containing GC and MZ cells were individually selected using the square tool from QuPath for every spleen section, condition, and layer. Layer alignment containing the same selected spot was performed on ImageJ using the TrackEM2 module. Next, color deconvolution was performed using 3-amino-9-ethylcarbazole–hematoxylin vectors, and a composite was created using channel 2 (detecting red, in ImageJ) for each stain. The composite was adjusted by inversion of the lookup table for each marker and given a pseudocolor. Segmentation and positive cell detection were performed using QuPath 0.3 software loading all images from the same time points and different genotypes to use the same parameters for all samples.

Every analyzed ROI containing a GC had a matched MZ ROI. Using QuPath software, we performed a segmentation analysis to identify individual cells using hematoxylin staining and default cell expansion (5 μm). We set up a threshold to discriminate positive from negative cells and obtain a value. Annotation measurements for every cell in the ROI were exported. The average for every ROI was calculated by total detection in the ROI.

### Signal normalization by number of detections per ROI

The normalized individual detection measurements for each ROI were calculated as follows: GCx+n represented the individual detection measurement for ROI GCx divided by the total number of detections. Similarly, MZx+n represented the individual detection measurement for ROI MZx divided by the total number of detections. An example comparison of these results using a paired 2-tailed *t* test involved sets of corresponding GCx+n and MZx+n values, such as GC1 paired with MZ1, GC2 paired with MZ2, GC3 paired with MZ3, and so forth through GCx+n and MZx+n.

### Mouse strains and immunizations

*Cγ1-cre* [*Ighg1tm1(cre)Cgn*] and *Atg7^fl^* (*Atg7tm1Tchi*) mice have been described previously (54, 55). Animals were housed in pathogen-free conditions with controlled day/night cycles. For T cell–dependent immunizations, 8- to 12-week-old mice were injected intraperitoneally with 100 μg NP-CGG (2BScientific) in aluminum adjuvant (Thermo Fisher Scientific). Spleens were collected at 7, 10, 21, and 35 days after immunization and processed for flow cytometry, GC B cell isolation, cell sorting, and histology analyses. All animal care and procedures were performed according to United Kingdom Home Office regulations under PPL license P68650650.

### Flow cytometry and GC B subset isolation

Homogenized splenic samples were resuspended in PBS plus 5% FCS, red blood cells lysed using RBC lysis buffer (BioLegend), and cells maintained at 4°C during the subsequent process. Splenocytes were stained with specific antibodies, washed, and analyzed by fluorescence-activated cell sorting (FACS) (LSR Fortessa, Becton Dickinson). Flow cytometry analysis was performed using FlowJo v10 software (Becton Dickinson). GC B cells were isolated using 2 sequential steps. The first step was performed using MACS Mouse Germinal Center B Cell (PNA) MicroBead Kit (Miltenyi Biotec). The PNA^+^ enriched fraction was then stained with anti-B220, anti-GL7, anti-CD95, and viability dye eFluor 780 (eBioscience). GC B subsets were then sorted by FACSAria Fusion Sorter (Becton Dickinson).

### Quantitative PCR

Total RNA was extracted from FACS-sorted splenic GC B cells isolated 10 days after immunization using the RNeasy Plus Mini Kit (QIAGEN), following the manufacturer’s protocol. RNA was quantified via Nanodrop and reverse-transcribed into cDNA using the High-Capacity cDNA Reverse Transcription Kit (Applied Biosystems/Thermo Fisher Scientific). Triplicate quantitative PCR (qPCR) reactions (15–50 ng input RNA) were set up with custom 10 μM primers, targeting LMNB1 and GAPDH (internal control), in 20 μL SYBR Green–based mixes (Thermo Fisher Scientific). qPCR was performed on an Applied Biosystems ABI QuantStudio 7.

LMNB1 probe amplification (by qPCR) used the following sequences: LMNB1 forward, 5′-GATCAGATTGCCCAGCTAGAA (custom made); LMNB1 reverse, 5′-CGAAACTCCAAGTCCTCAGTAA (custom made). GAPDH probe amplification (by qPCR) used the following sequences: GAPDH forward, 5′-GGGTGTGAACCACGAGAAATA (custom made); GAPDH reverse, 5′-GTCATGAGCCCTTCCACAAT (custom made).

### ELISA

Blood was extracted from the tail vein on days 4, 7, 10, and 21 after NP-CGG immunization and kept at 4°C overnight. Whole blood was centrifuged at 12,000*g* for 5 minutes, and the serum was collected. Samples were diluted 1 in 16,000 and added to 96-well plates (50 μL per well) precoated with 10 μg/mL NP_27_-BSA (Biosearch Technologies) or 10 μg/mL NP_9_-BSA (Biosearch Technologies). A standard curve for quantitative ELISA was performed to determine IgG1 concentration (ng/μL). Bound antibodies were revealed by detection with alkaline phosphatase–conjugated anti-mouse IgG1 (Southern Biotech). *Cγ1Cre^+/–^* samples from an average of 4 days after immunization were used as control.

### GSEA

We used human and mouse datasets extracted from Victora et al. (23). Gene sets for autophagy signatures were obtained from the Molecular Signatures Database (MSigDB) and the Reactome database and analyzed using GSEA software (http://www.broadinstitute.org/gsea/index.jsp) with the specific settings: permutations, 1,000; permutation type, gene set; metric for ranking genes, *t* test.

For human datasets, we selected the following autophagy signatures: GOBP_NEGATIVE_REGULATION_OF_AUTOPHAGY.v2023.1.Hs; GOBP_REGULATION_OF_AUTOPHAGY.v2023.1.Hs; REACTOME_AUTOPHAGY.v2023.1.Hs; REACTOME_SELECTIVE_AUTOPHAGY.v2023.1.Hs.

For mouse datasets, we selected the following autophagy signatures: GOBP_NEGATIVE_REGULATION_OF_AUTOPHAGY.v2023.1.Mm; GOBP_REGULATION_OF_AUTOPHAGY.v2023.1.Mm; REACTOME_SELECTIVE_AUTOPHAGY.v2023.1.Mm.

### scRNA-Seq and snATAC-Seq sample preparation

For scRNA-Seq, 3–4 mice per condition (*Cγ1Cre^+/–^* and *Cγ1Cre Atg7^fl/fl^*) were immunized with NP-CGG and culled at 2 different time points (10 and 21 dpi). To enrich the sample with GC B cells, cells were isolated using PNA MACS and then sorted as described above. Viability greater than 85% and an optimal input cell concentration of 1,200 cells/μL were used.

We enriched the GC B cell fraction using a 2-step process. First, the PNA^+^ fraction was isolated using a MACS isolation kit (Miltenyi Biotec). Second, the PNA^+^ fraction was labeled with antibodies and sorted based on VDJ region–negative, B220^+^, GL7^+^, and CD95^+^ cells. Cells were collected in collection buffer (1× PBS, 0.04% UltraPure BSA [50 mg/mL]) and GC B cells mixed with total splenocytes in a 1:3 ratio. For scRNA-Seq and snATAC-Seq experiments, 0.25 × 10^6^ GC B cells and 0.75 × 10^6^ splenocytes were mixed.

### scRNA-Seq library preparation

Cells at 1,200 cells/μL were processed using a 10x Genomics Chromium Single-Cell 3′ Reagent kit v3.1 (dual index) and individually barcoded with a 10x Genomics Chromium controller. scRNA-Seq libraries were sequenced on an Illumina NextSeq 550 with NextSeq 500/550 High Output Kit v2.5 (75 cycles; 20024906, Illumina) using an Illumina NovaSeq 6000 instrument.

### scRNA-Seq data analysis

The sequenced 10x Genomics Chromium libraries from 4 samples were mapped to the mm10 mouse genome and assigned to droplets with Cell Ranger software (version 6.0.1) with default parameters. Transcriptomes of 24,886 cells with a median unique molecular identifier (UMI) count of 2,437 per cell and 35,571 cells with a median UMI count of 2,253 per cell were obtained for days 10 and 21, respectively. To obtain the individual cell embeddings at day 10 and day 21, the resulting read count matrices were analyzed with Seurat (version 3.0.2) (56).

Only cells where the number of detected genes was higher than 400 and lower than 8,000 and the percentage of reads mapped to mitochondrial genes was less than 10% were included in analysis.

To identify cell doublets, DoubletFinder (version 3) (57) was applied to *Cγ1Cre^+/–^* 10 dpi (Control), *Cγ1Cre Atg7^fl/fl^* 10 dpi (Exp), *Cγ1Cre^+/–^* 21 dpi (C21), and *Cγ1Cre Atg7^fl/fl^* 21 dpi (E21) RData objects separately.

Seurat objects were integrated using canonical correlation analysis, and SCTransform was applied for normalization. Variable features were identified using the FindVariableFeatures function, and data were scaled before dimensionality reduction. Clustering and visualization followed the standard Seurat workflow. Cluster markers were identified using FindAllMarkers at 0.5 resolution. Uploading the DEGs per cluster to the curated database CellKB (58), we named clusters in our scRNA-Seq integrated dataset.

#### DEG analysis.

The FindMarkers function was used to identify DEGs. Default thresholds were applied: 0.1 for the minimum percentage of cells expressing a gene and 0.25 for minimum log fold change. DEGs were filtered using adjusted *P* values to control the false discovery rate (FDR), with significance defined as adjusted *P* less than 0.05. The Benjamini-Hochberg method was used for *P* value adjustment.

#### GSEA and GO.

Genes were ranked based on the statistical metrics log fold change (>1) and adjusted *P* value less than 0.05. The input for the Gene Set Enrichment Analysis (gseGO) function using the clusterProfiler package was a ranked list of the DEGs. Predefined gene sets based on GO terms were assessed for significant enrichment among the identified DEGs. Dotplot (https://ggplot2.tidyverse.org) was used to visualize the top 10 GO terms.

#### Module score analysis.

The AddModuleScore function was used in the Seurat environment to summarize the activity or expression of groups of genes (gene modules) within individual cells.

#### Cell cycle analysis.

The CellCycleScoring function in Seurat was used for cell cycle assessment in the merged dataset. Gene sets associated with S and G_2_/M phase transitions were sourced from ref. 59.

#### Trajectory and pseudotime analysis.

DEGs were used to perform the trajectory and pseudotime analysis using Monocle (version 2.0) (60). Custom-made code was generated for the integrated object. Differences between conditions at 10 and 21 dpi time points were visualized using dimensionally reduced Component_1 and Component_2. Genes that changed as a function of pseudotime were plotted using a heatmap and cluster separation.

#### RNA velocity analysis.

Exonic and intronic gene counts in BAM files from Cell Ranger were analyzed using Velocyto (version 0.17.13). The repeat regions of the genome were masked. Loom files were analyzed with scvelo (version 0.1.24) (61). The dynamic model was used to estimate the velocities, and the estimated velocity field was plotted on top of the UMAP embeddings.

### snATAC-Seq library preparation

The splenocyte mix was pelleted by centrifugation (300*g*, 5 minutes, 4°C), the supernatant was removed, and cells were resuspended in 50–100 μL 0.04% BSA–PBS buffer. Cells were centrifuged (300*g*, 5 minutes, 4°C), the supernatant carefully removed, and 100 μL of 1× chilled nucleus lysis buffer (10 mM Tris-HCl, pH 7.4, 10 mM NaCl, 3 mM MgCl_2_, 0.1% Tween 20, 0.1% NP-40, 0.01% digitonin, 1% BSA in nuclease-free water) added to the mix. Lysis was performed for exactly 3 minutes at 4°C, followed by the addition of 1 mL washing buffer (10 mM Tris-HCl, pH 7.4, 10 mM NaCl, 3 mM MgCl_2_, 1% BSA, 0.1% Tween 20 in nuclease-free water). After centrifugation (300*g*, 5 minutes, 4°C), the supernatant was removed, and 200 μL of chilled diluted nucleus buffer (2000207, 10x Genomics) was added. Nuclei were counted and centrifuged again (300*g*, 5 minutes, 4°C) and later diluted in a chilled dilution buffer to obtain a targeted nuclei recovery of 10,000 cells. The transposition mix was prepared with 3 μL of nuclei suspension based on the manufacturer’s protocol. Transposition was performed for 1 hour at 37°C, followed by supplementation of master mix and beads (Single Cell ATAC Gel Beads v1.1 and reagents, 1000175, 10x Genomics), loading on 10x Chromium Next GEM Chip H (1000161, 10x Genomics), and processing on a 10x Chromium Controller (120212, 10x Genomics). GEM incubation was performed with 12 cycles of PCR. The library was prepared according to the protocol with cycle numbers dependent on input nuclei concentration. snATAC libraries were sequenced on an Illumina NextSeq 550 with NextSeq 500/550 High Output Kit v2.5 (75 cycles; 20024906, Illumina).

### snATAC-Seq data analysis

Raw sequencing data were pre-processed using the Cell Ranger ATAC v.1.1.0 pipeline (10x Genomics).

Peaks from *Cγ1Cre^+/–^* 10 dpi (Control) and *Cγ1Cre Atg7^fl/fl^* 10 dpi (Exp) samples were filtered based on length (keeping only peaks more than 20 bp and less than 10,000 bp) and annotated to the reference from Cell Ranger refdata-cellranger-arc-mm10-2020-A-2.0.0. Quality metrics for the snATAC-Seq were obtained from the Cell Ranger ATAC output. We merged filtered and annotated files in a single object for normalization, dimensionality reduction, cell clustering, and finding of differential accessibility regions.

#### Differentially accessible peaks.

Differential chromatin accessibility between cell types was assessed with Signac (version 1.6.0) (62). Peaks were detected in at least 10% of cells using a likelihood ratio test and a log fold change threshold of 0.25. Bonferroni-corrected *P* values were used to determine significance at an FDR less than 0.05. Genomic regions containing snATAC-Seq peaks were annotated with clusterProfiler (version 3.16.1) (63) using the UCSC database (64) on mm10. To annotate peaks to the 4 categories (exon, intergenic, intron, and promoter–transcription start site [TSS]), the accessible regions were scanned using annotatePeaks.pl from HOMER (http://homer.ucsd.edu/homer/ngs/annotation.html). Common peaks in graphs were observed in at least 1% of cells in both conditions. For the variable peaks, the cutoff was set by the addition of a condition of fold change ≥ 10 >10 (65).

#### GO in DA peaks.

601 DA peaks (*P* < 0.05) were overlapped to 32,285 genes from 10x Genomics annotation (refdata-cellranger-arc-mm10-2020-A-2.0.0), which was also used in our snATAC-Seq analysis.

DA up- and downregulated peaks from *Cγ1Cre Atg7^fl/fl^* samples were separately analyzed using the enrichGO function from the clusterProfiler R package (version 4.12.6).

### Software and algorithms

We used GraphPad Prism 9.0; QuPath v2.0-m8 and v3.0; FlowJo v10; ImageJ; Fiji (ImageJ version 1.54p); Basic Local Alignment Search Tool (BLAST; NCBI); IMGT/V-QUEST (https://www.imgt.org/IMGT_vquest/); MetaMorph; Zeiss ZEN, black edition; and R Studio.

### Statistics

Statistical analyses were performed using GraphPad Prism (version 9) software. Two-tailed unpaired Student’s *t* test analysis was performed to compare 2 experimental groups. A variation of this test, the 2-tailed paired Student’s *t* test, was used to compare the same condition in 2 different situations. No corrections were applied unless otherwise stated. To compare 3 or more matched groups, we used repeated-measures 1-way ANOVA followed by Tukey’s post hoc test, unless otherwise stated in the figure legend. In all figures, bars represent the mean ± SEM. A *P* value of less than 0.05 was considered statistically significant.

### Study approval

All experiments involving animals were approved by the Queen Mary University Animal Welfare and Ethical Review Board and performed under the United Kingdom Home Office license No. P68650650. Reactive tonsils and lymph nodes were obtained from routine lymphaden- and tonsillectomies performed at the St. Bartholomew Hospital, London, United Kingdom. All samples were the subject of Queen Mary Ethics of Research Committee of Queen Mary University of London approval and were obtained according to the Human Tissue Authority license and regulations (HTA license 12199). Sex was not considered as a biological variable. The demographic, clinical, and histological patient characteristics are summarized in Supplemental Table 1.

Labial salivary gland biopsies were collected after informed consent from patients with a confirmed diagnosis of Sjögren’s disease, according to the 2016 American College of Rheumatology/European League Against Rheumatism classification criteria. The study was approved by the Queen Mary Ethics of Research Committee of Queen Mary University of London (REC 05/Q0702/1-Rheumatology/Oral Medicine Clinic). The demographic, clinical, and histological patient characteristics are summarized in Supplemental Table 8.

For ex vivo B cells, anonymized leukocyte cones were obtained from healthy adult donors attending platelet donation clinics at the Southampton Blood Donor Centre (National Blood Service, Southampton, United Kingdom). Ethical approval was provided locally by the University of Southampton Faculty of Medicine Ethics Committee (19660.A11) and nationally by the National Health Service/Health and Social Care Research Ethics Committee (IRAS: 186605).

### Data availability

The primary sequence read files for scRNA-Seq and snATAC-Seq experiments were deposited to the National Center for Biotechnology Information Gene Expression Omnibus database (accession GSE218052). scRNA-Seq and snATAC-Seq analysis and figure generation were obtained using publicly available codes for R Studio with minor modifications. Custom-made codes were generated for trajectory and pseudotime analysis and can be accessed via GitHub (https://github.com/braunslab?tab=repositories; commit IDs 1903998 and 848c65f).

Values for all data points in graphs can be found in the Supporting Data Values file.

## Author contributions

AB, TK, and MCS conceived and designed the study. AB, TK, JGG, MSC, and DPC supervised the study. MCS, MC, SJ, AC, MTB, and FF performed the experiments. JO, MB, and EP oversaw the sourcing, compliance, and usage of clinical samples. MCS, CHS, GB, and KYY analyzed the scRNA-Seq and snATAC-Seq data. MCS, AB, and TK wrote the manuscript. All authors contributed to the final version of the manuscript.

## Supplementary Material

Supplemental data

Unedited blot and gel images

Supplemental tables 1-9

Supporting data values

## Version 1 05/21/2025

In-Press Preview

## Version 2 07/01/2025

Electronic publication
