# Supplementary material for: Autophagy is an upstream mediator of chromatin dynamics in normal and autoimmune germinal center B cells
Source: J Clin Invest. 2025 May 15;135(13):e178920. doi: 10.1172/JCI178920 (PMC12208547; doi:10.1172/JCI178920)
Supplement: Supplemental data [file jci-135-178920-s182.pdf]

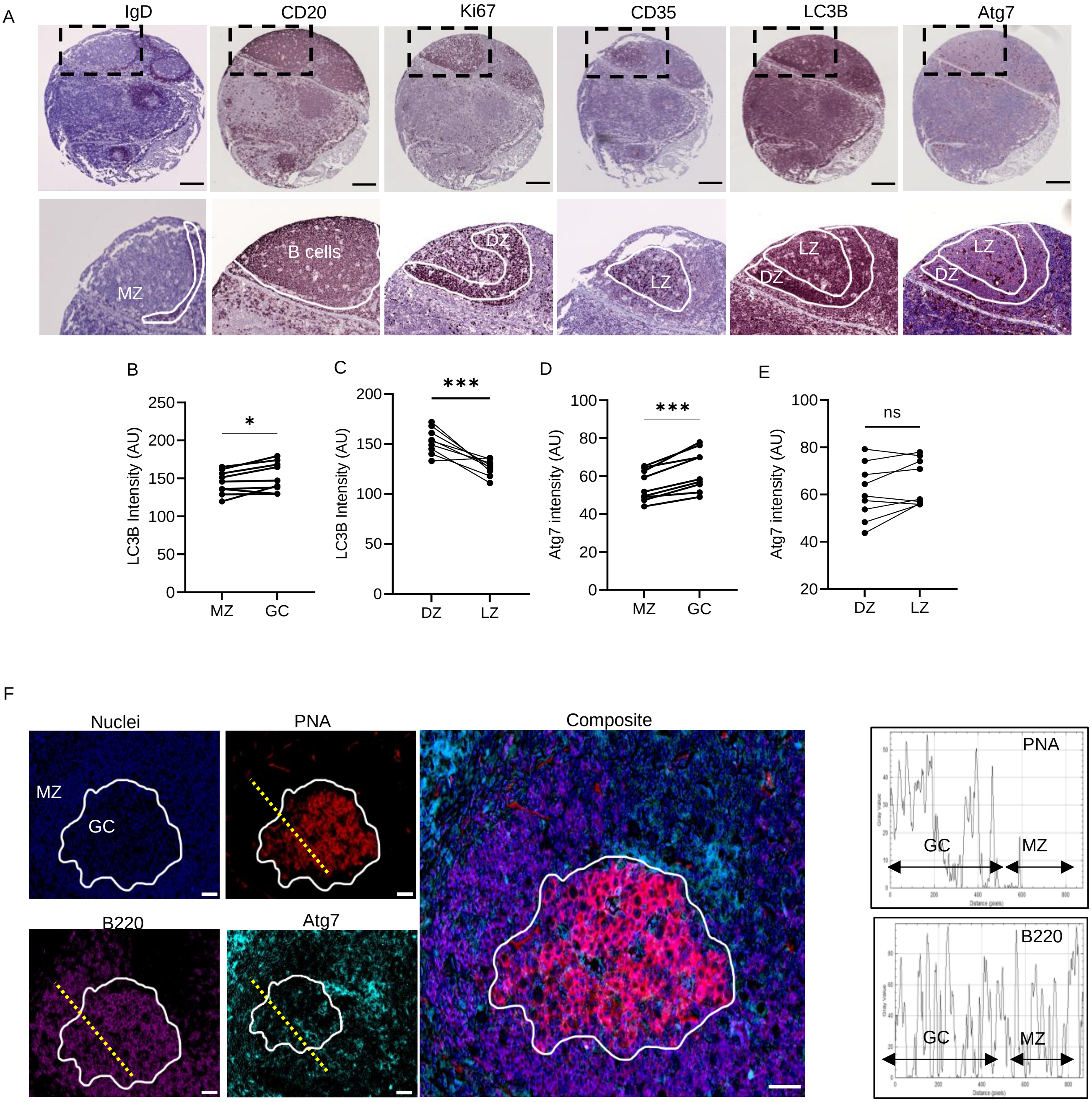

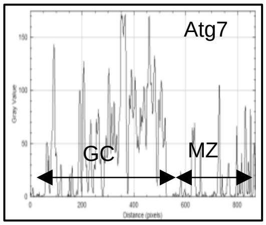

Supplemental Figure 1. Autophagy is enhanced in GC's DZ.

A. Representative images obtained from multiplex immunohistochemistry on human follicles (delineated a GC) from reactive lymph nodes. Lymph nodes were stained with anti-IgD, anti-CD20, anti-Ki67, anti-CD35, anti-LC3B and anti-Atg7 antibodies, and nuclei were counterstained with haematoxylin (blue). Scale bar 500 µm. B. LC3B intensity was compared in two follicular areas (MZ and GC). At least 3 GCs per patient were analysed from a total of nine patients. *p<0.05; paired two-tailed Student's t-test. C. LC3B intensity was compared in DZ and LZ areas. ** p<0.001; paired two-tailed Student's t-test. D. Atg7 intensity was compared in two follicular areas (MZ and GC). At least 3 GC /patient were analysed from a total of nine patients. *** p<0.001; paired two-tailed Student's t-test. E. Atg7 intensity was compared in DZ and LZ areas. Paired two-tailed Student's t-test. F. Representative images obtained from multiplex immunohistochemistry on mouse spleen 10 d.p.i. Line-scans showing PNA, B220 and Atg7 expression in the GC and MZ. This experiment was conducted on at least three mice in three independent immunisations.

Supplemental Figure 2

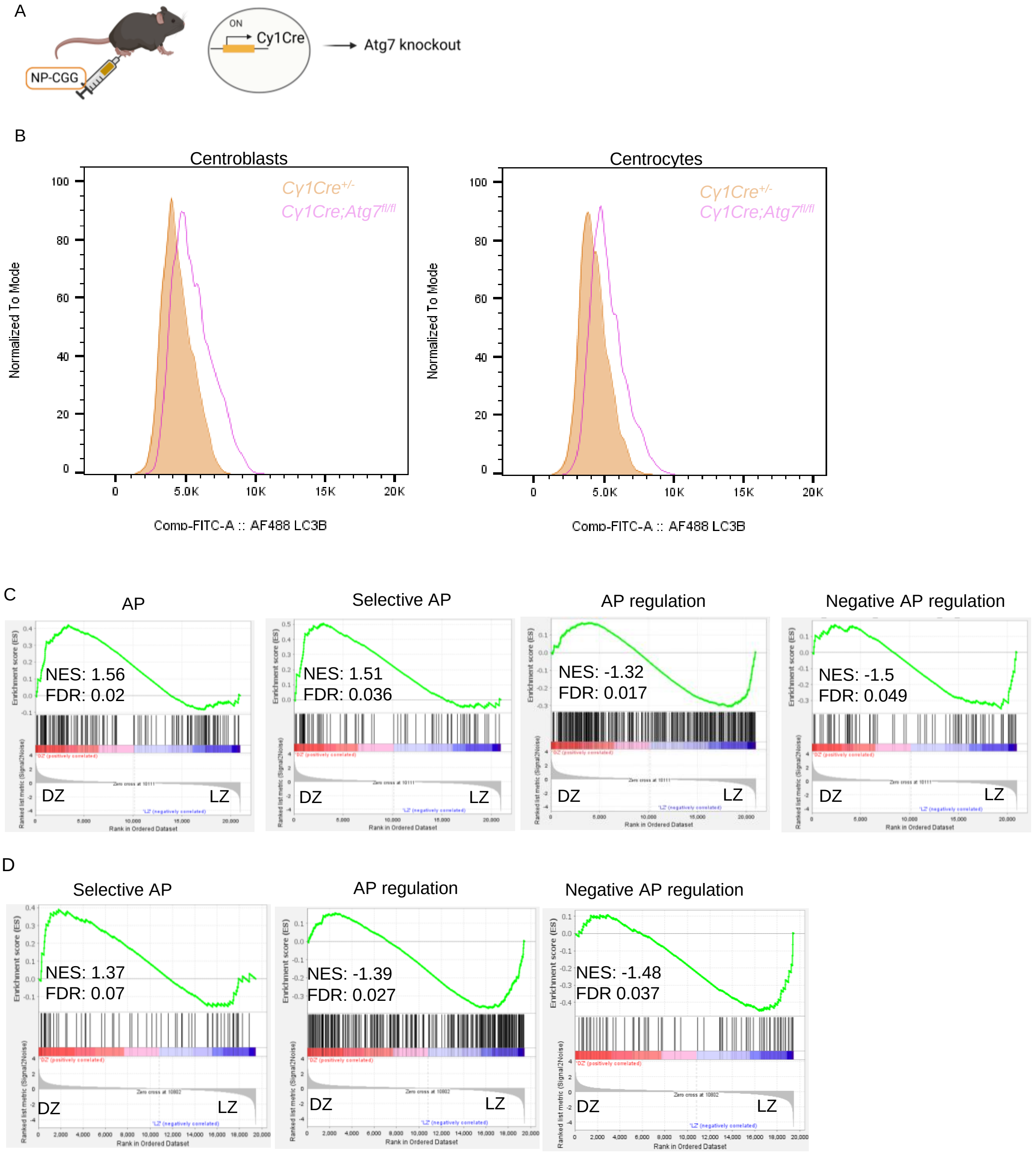

Supplemental Figure 2. Centroblasts are the GC B cells that exhibit the highest levels of autophagy.

A. Schematic representation of the inducible Atg7 knockout mouse model. B. Distribution of LC3B GC B cells among LZ (centrocytes) and DZ (centroblasts) subsets, assessed by flow cytometry comparing *Cγ1Cre+/-* and *Cγ1Cre;Atg7fl/fl*. C. Gene Set Enrichment Analysis (GSEA) performed using Human dataset (GSE38697) extracted from Victora et al., 2012 and analysed for signatures obtained from MSigDB “Autophagy”, “Selective autophagy”, “Autophagy regulation” and “Negative autophagy regulation”. D. GSEA performed using mouse dataset (GSE38712) extracted from Victora et al., 2012 and analysed for signature obtained from MSigDB “Selective autophagy”, “Autophagy regulation” and “Negative autophagy regulation”.

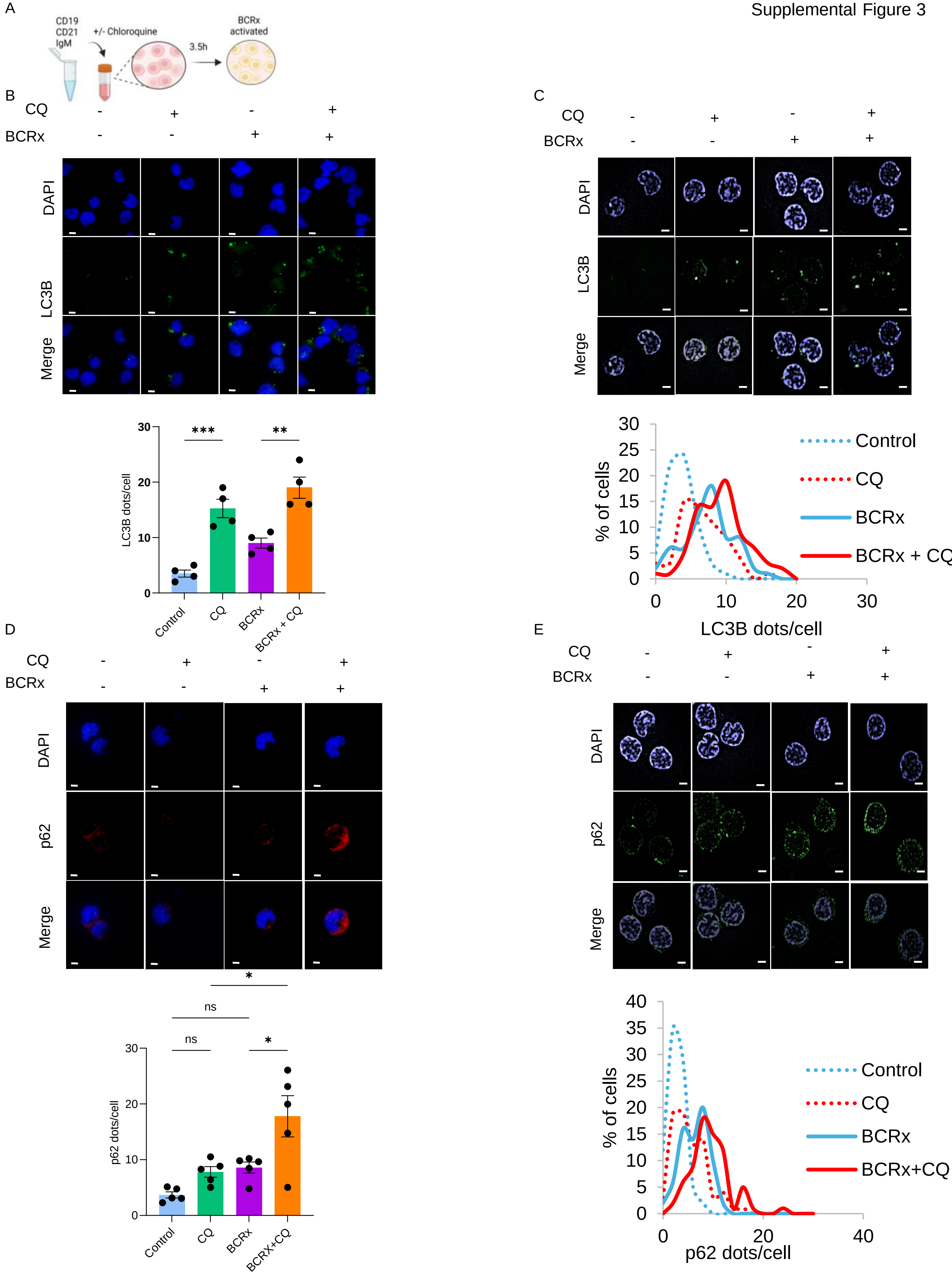
A
CD19
CD21
IgM
+/- Chloroquine
3.5h
BCRx
activated
B
CQ - + - +
BCRx - - + +
DAPI
LC3B
Merge
LC3B dots/cell
***
**
Control
CQ
BCRx
BCRx + CQ
C
CQ - + - +
BCRx - - + +
DAPI
LC3B
Merge
% of cells
LC3B dots/cell
Control
CQ
BCRx
BCRx + CQ
D
CQ - + - +
BCRx - - + +
DAPI
p62
Merge
*
ns
ns
*
p62 dots/cell
Control
CQ
BCRx
BCRX+CQ
E
CQ - + - +
BCRx - - + +
DAPI
p62
Merge
% of cells
p62 dots/cell
Control
CQ
BCRx
BCRx+CQ

Supplemental Figure 3. Increased LC3B and p62 puncta in BL2 cell line and human peripheral B cells upon BCR engagement.

A. Schematic representation of the in vitro experiment to stimulate BCR in the BL2 cell line and human peripheral B-cells. B. Representative images from LC3B immunofluorescence upon BL2 BCR engagement +/- 10µM chloroquine. Below is the LC3B quantification (n=4 experiments). Two-way ANOVA with multiple comparisons. C. Human peripheral B-cells upon BL2 BCR engagement +/- 10µM chloroquine. Below is the LC3B dots histogram (n=2 experiments). D. Representative images from p62 immunofluorescence upon BL2 BCR engagement +/- 10µM chloroquine. Below is the p62 dots quantification (n=5 experiments). Two-way ANOVA with multiple comparisons. E. Human peripheral B-cells upon BL2 BCR engagement +/- 10µM chloroquine. Below p62 dots histogram (n= 2 experiments)

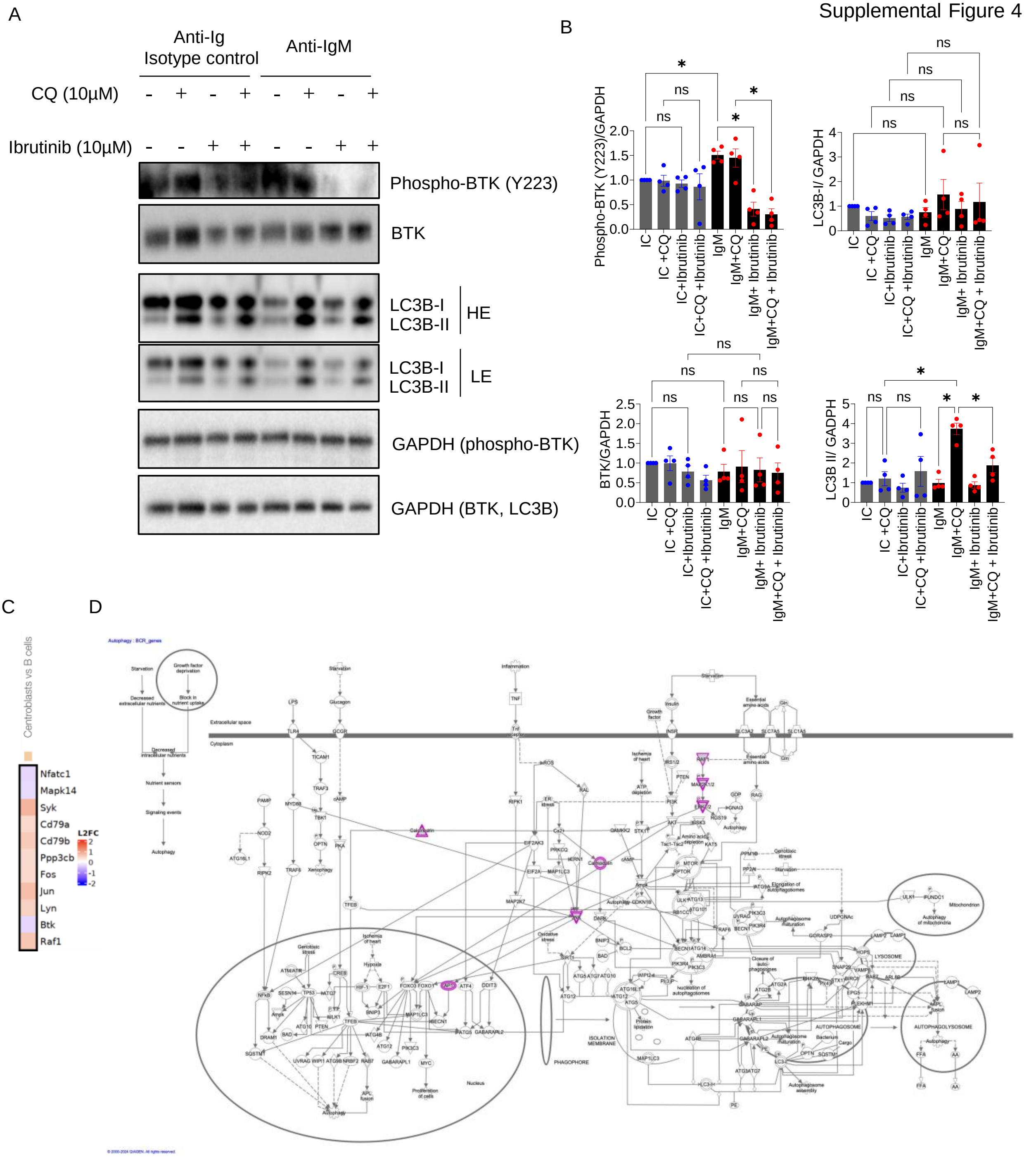

Supplemental Figure 4. BCR is an upstream activator of autophagy in activated B cells.

A. Representative immunoblot analysis of BL2 cells upon IgM stimulation combined with chloroquine (autophagy inhibitor) and Ibrutinib (BCR signalling inhibitor). B. Immunoblot quantifications of phospho-BTK (Y223), BTK, LC3B II and LC3B I from 4 biologically independent experiments. C. Heatmap showing only significant comparisons (adjusted p value<0.05) between centroblasts vs B cells for the 31 genes involved in the BCR pathway at the peak of the GC reaction (10 d.p.i). D. Autophagy IPA pathway highlighting in purple the BCR upregulated genes in centroblasts after 10 d.p.i.

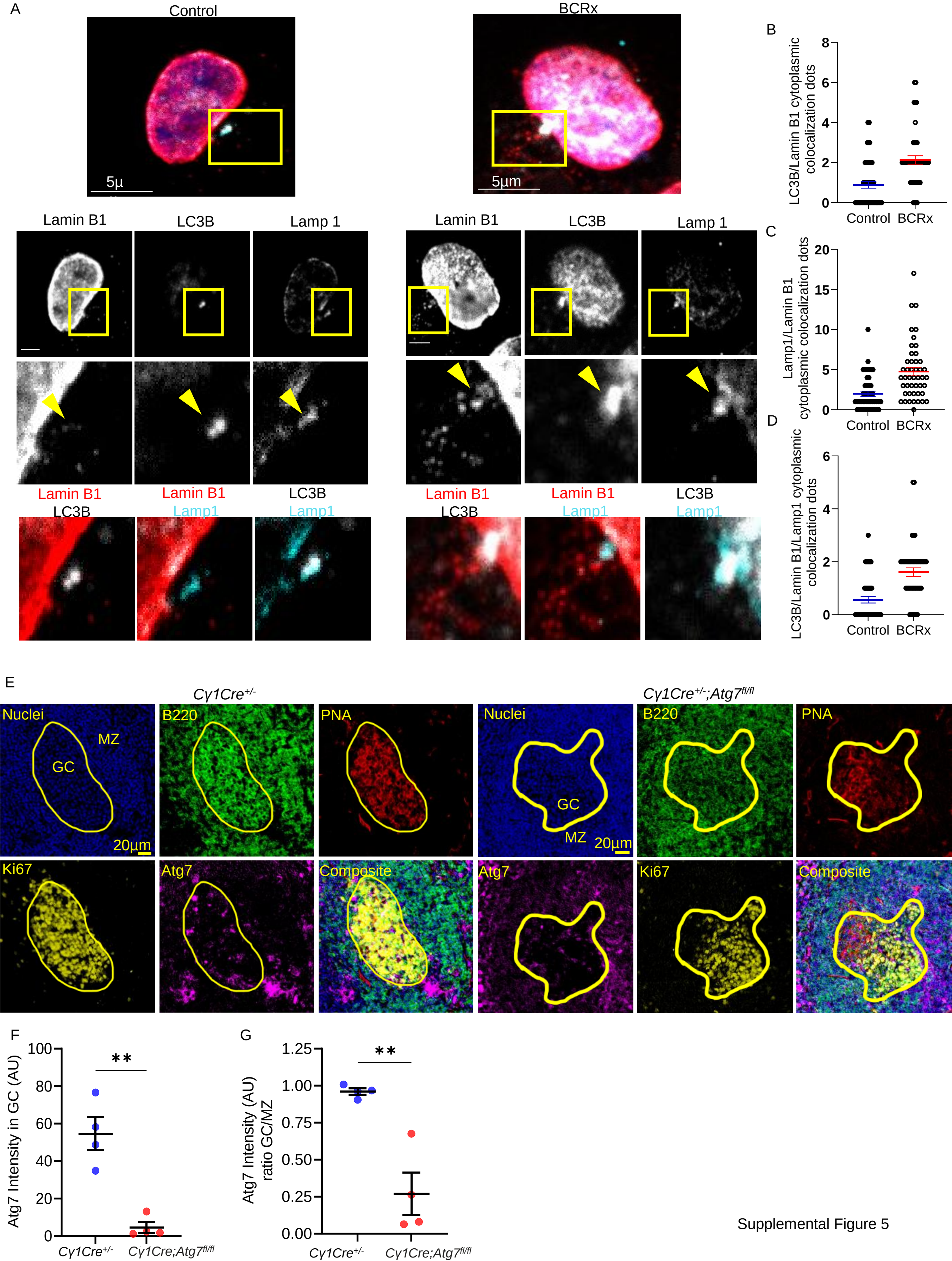

Supplemental Figure 5

Supplemental Figure 5. Lamin B1 and autophagy machinery interaction.

A. BCR was cross-linked (BCRx) for 3.5h in the BL2 cell line and stained with anti-Lamin B1 (red), anti-LC3B (grey) and anti-Lamp1 (cyan) antibodies. Images were taken using confocal microscopy LSM880 Oil-63X + 4.0 zoom and cytoplasmic dots were quantified in two independent experiments n=46 cells. B. Quantification of LC3B and Lamin B1 cytoplasmic colocalisation dots from (A). C. Quantification of Lamp1 and Lamin B1 cytoplasmic colocalisation dots from (A). D. Quantification of LC3B, Lamin B1 and Lamp1 cytoplasmic colocalisation dots from (A). E. Multiplex IHC showing pseudo-coloured nuclei (Blue), B220 (green), PNA (red), Ki67 (Yellow) and Atg7 (Magenta) in representative GC from *Cγ1Cre+/-* and *Cγ1Cre;Atg7fl/fl* mouse spleen sections. F. Atg7 intensity (AU) measured at least 3GC/mouse in n=4 mice from 2 independent experiments. Horizontal bar represents the mean ± SEM. ** $p<0.001$. two-tailed Student's t-test. Images shown were previously presented in Figure 2F and are reproduced here for extended analysis. G. Atg7 intensity (AU) ratio between GC/ MZ measured in at least 3 follicles/ mouse in n=4 mice from 2 independent experiments. Horizontal bar represents the mean ± SEM. ** $p<0.001$. two-tailed Student's t-test.

Supplemental Figure 6

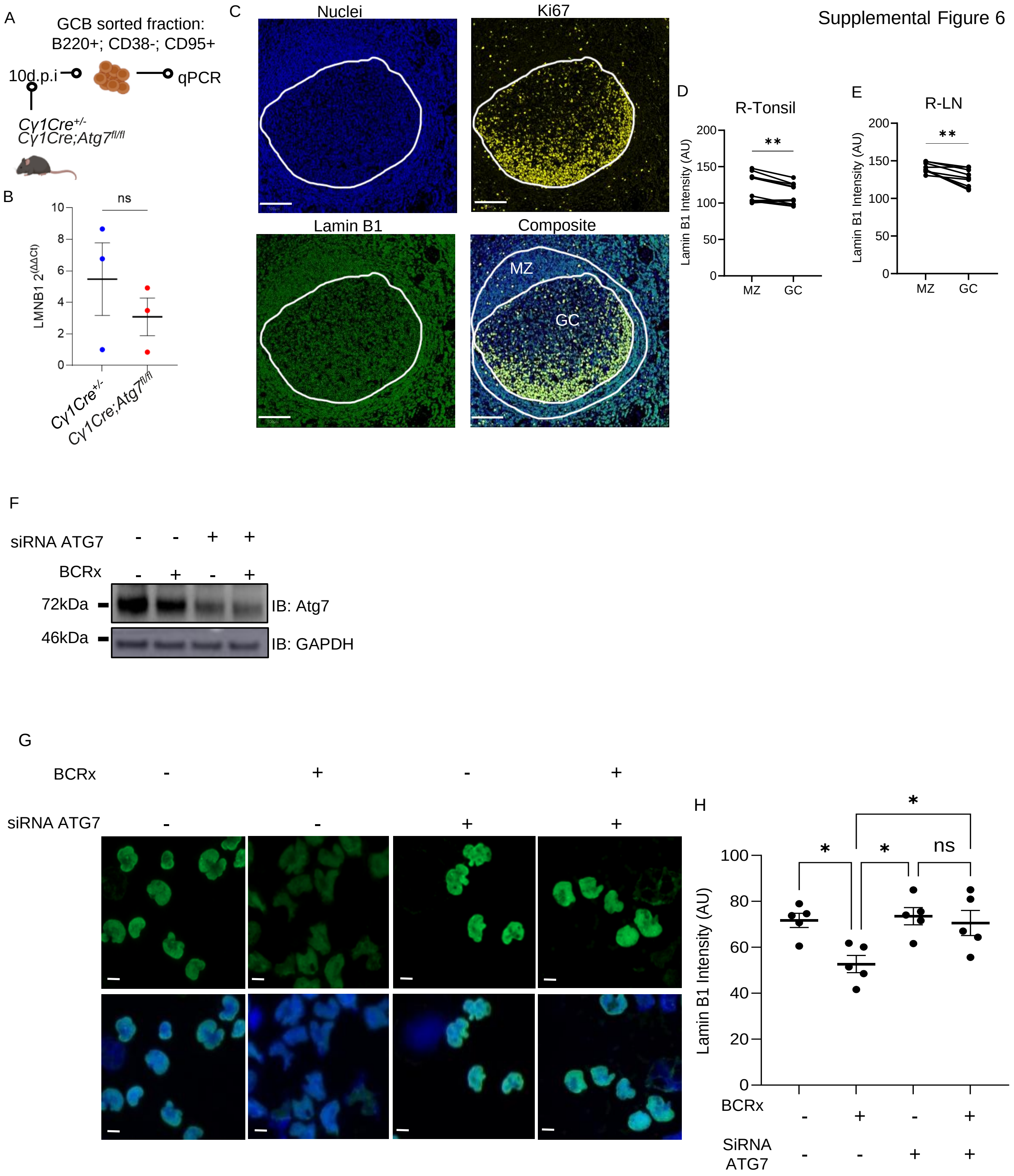

Supplemental Figure 6. Lamin B1 is regulated at post-transcriptional level.

A. Experimental design of the qPCR assay in mouse GC B cells. B. Relative LMNB1 expression levels of in *Cγ1Cre +/-* and *Cγ1Cre;Atg7fl/fl* mice. C. Representative multiplex immunohistochemistry image of human GC from a reactive tonsil. Tonsils and Lymph nodes were stained with anti-Ki67 (Yellow) and anti-Lamin B1 (Green) antibodies, and nuclei were counterstained with haematoxylin (Blue). Scale bar 500 µm. D. Lamin B1 intensity was compared in two follicular areas (MZ and GC). 3 to 6 GCs per tonsil were analysed from N=3 patients. ** $p<0.001$; paired two-tailed Student's t-test. E. Lamin B1 intensity was compared in two follicular areas (MZ and GC) per lymph node. At least 3 GC/patient were analysed from a total of nine patients. ** $p<0.001$; paired two-tailed Student's t-test. F. Atg7 Western Blot analysis performed in RNAi Atg7 +/- BCRx in BL2 cells. Representative result of three independent experiments. G. Representative Lamin B1 immunofluorescence 3.5 hours after BCR cross-linking (BCRx) in BL2 cells co-treated with Atg7 siRNA. Scale bar 15µm. H. Lamin B1 fluorescence intensity quantification. Five independent experiments were analysed. Bars are the mean ± SEM. **** $p<0.0001$. A two-way ANOVA test with multiple comparisons was performed.

Supplemental Figure 7

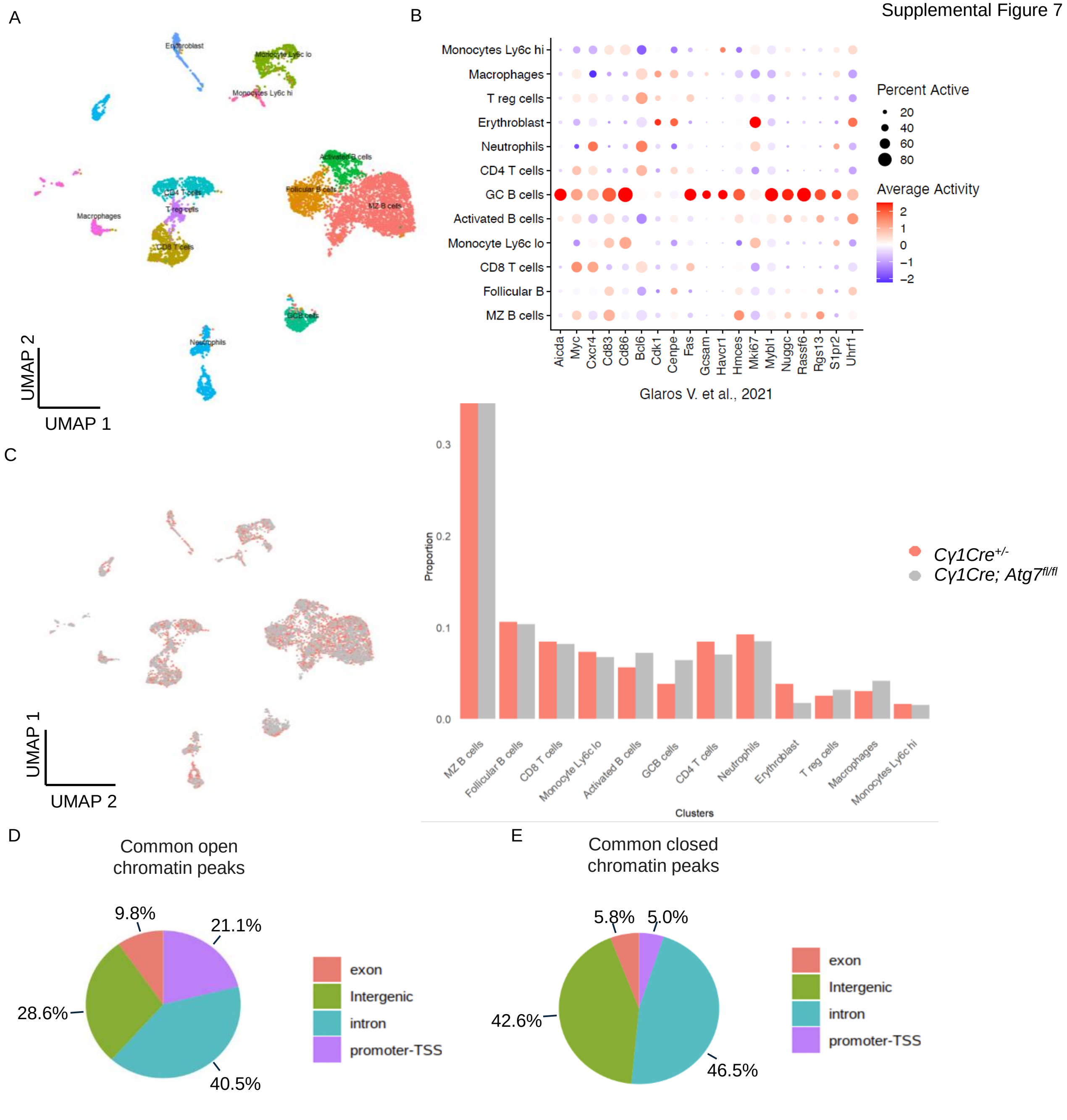

Supplemental Figure 7. snATAC-seq in *Cγ1Cre+/-* and *Cγ1Cre;Atg7fl/fl* splenocytes.

A. UMAP visualisation of splenocytes analysed by snATAC-seq for *Cγ1Cre+/-* and *Cγ1Cre;Atg7fl/fl* samples at 10 d.p.i. B. Dotplot showing GC signature extracted from Glaros et al., 2021. C. Individual UMAP plot visualisation per genotype (left). Barplot showing cell proportion per cluster and experimental genotypes in snATAC-seq data (right). D-E. Pie chart detailing the distribution of common open (D) and closed (E) chromatin peaks (Exon, Intergenic, Intron and Promoter-TSS) found in GC B cell cluster in *Cγ1Cre+/-* and *Cγ1Cre;Atg7fl/fl*.

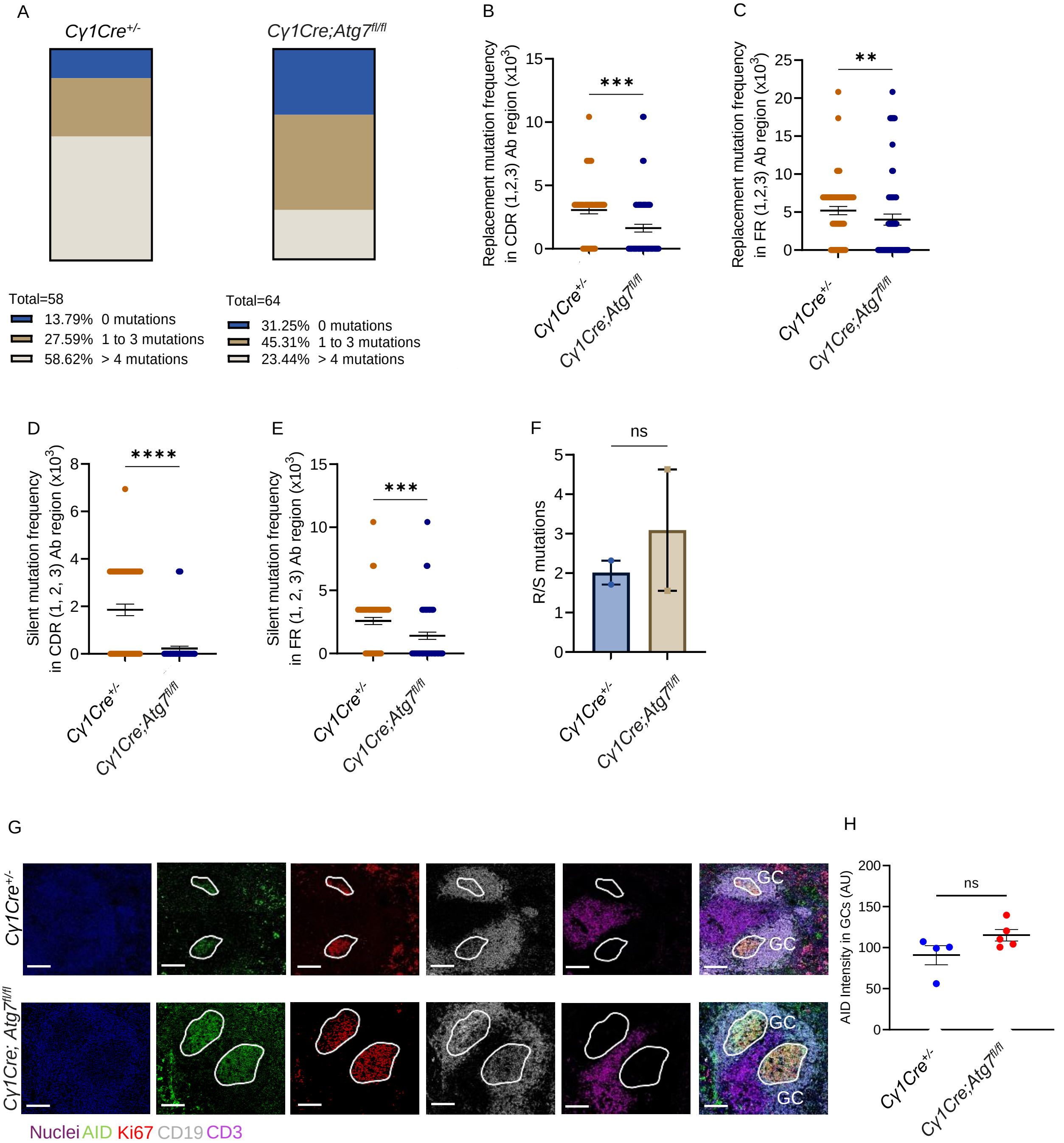

Supplemental Figure 8. Reduced mutational load in autophagy-impaired GC B cells.
A. Stack charts showing the fraction of Vh186.2 sequences loaded with a different number of mutations in sorted GC B cells of *Cγ1Cre+/-* and *Cγ1Cre;Atg7fl/fl* mice at 10 d.p.i. N=2 mice were analysed per genotype. B-C. Replacement mutation frequency in CDR (C) and FR (D) in Vh186.2 IgV region from sorted GC B cells of *Cγ1Cre+/-* and *Cγ1Cre;Atg7fl/fl* mice at 10 d.p.i. D-E. Silent mutation frequency in CDR (E) and FR (F) in Vh186.2 IgV region from sorted GC B cells of *Cγ1Cre+/-* and *Cγ1Cre;Atg7fl/fl* mice, 10 d.p.i. Unpaired T-test coupled to Mann-Whitney. F. Ratio Replacement versus Silent mutations in Vh186.2 IgV region from sorted GC B cells of *Cγ1Cre+/-* and *Cγ1Cre;Atg7fl/fl* mice at 10 d.p.i. Unpaired T-test. G. Representative QuPath pseudocoloured images obtained from multiplex immunohistochemistry. Spleen cuts were stained with anti-AID (green), anti-Ki67 (red), anti-CD19 (grey), and anti-CD3 (magenta) antibodies. Nuclei were counterstained with haematoxylin (blue). Scale bar 200 µm. H. AID intensity (AU) measured in at least 3 GC/mouse from two independent immunisations. n=4 mice in *Cγ1Cre+/-* and n=5 mice in *Cγ1Cre;Atg7fl/fl* were used for the analysis. Horizontal bars represent the mean ± SEM. Unpaired t-test.

Supplemental Figure 9

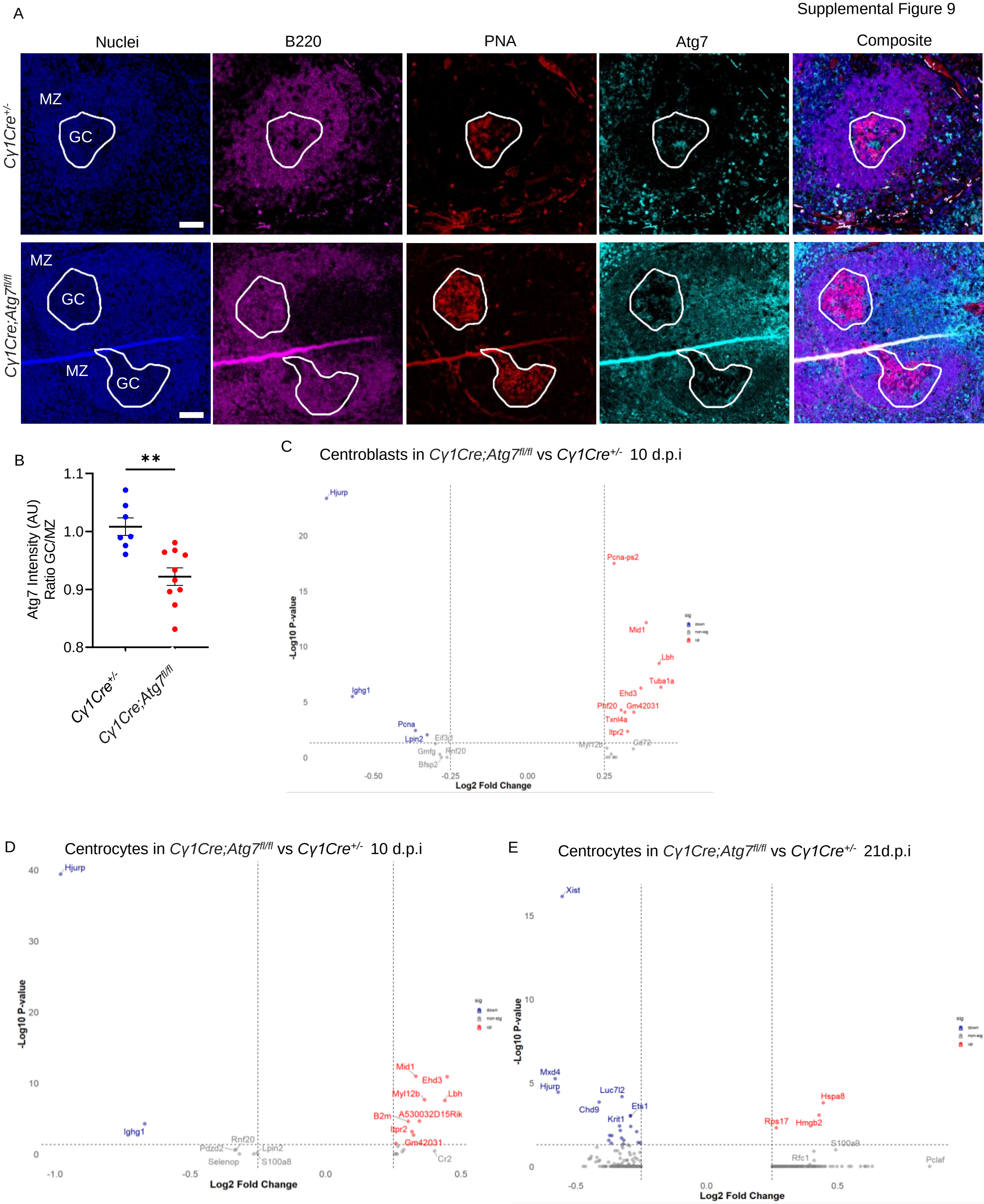

Supplemental Figure 9. Atg7 deletion in GC B cells.

A. Representative QuPath pseudocoloured images obtained from multiplex immunohistochemistry of *Cγ1Cre+/-* vs *Cγ1Cre;Atg7fl/fl* GC samples at 21 d.p.i. Spleens were stained with anti-B220 (magenta), anti-PNA (red) and anti-Atg7 (cyan) antibodies, and nuclei were counterstained with haematoxylin (blue). Scale bar 500 μm. B. Atg7 intensity (AU) ratio between GC/ MZ measured in at least three follicles/mouse in n=4 mice from 2 independent experiments. Horizontal bar represents the mean ± SEM. Unpaired T-test. C. Volcano plot showing centroblasts DEG comparing *Cγ1Cre;Atg7fl/fl* vs *Cγ1Cre+/-* genotypes at 10 d.p.i. D. Volcano plot showing centrocytes DEG comparing *Cγ1Cre; Atg7fl/fl* vs *Cγ1Cre+/-* samples at 10 d.p.i. E. Volcano plot showing centrocytes DEG comparing *Cγ1Cre; Atg7fl/fl* vs *Cγ1Cre+/-* samples at 21 d.p.i.

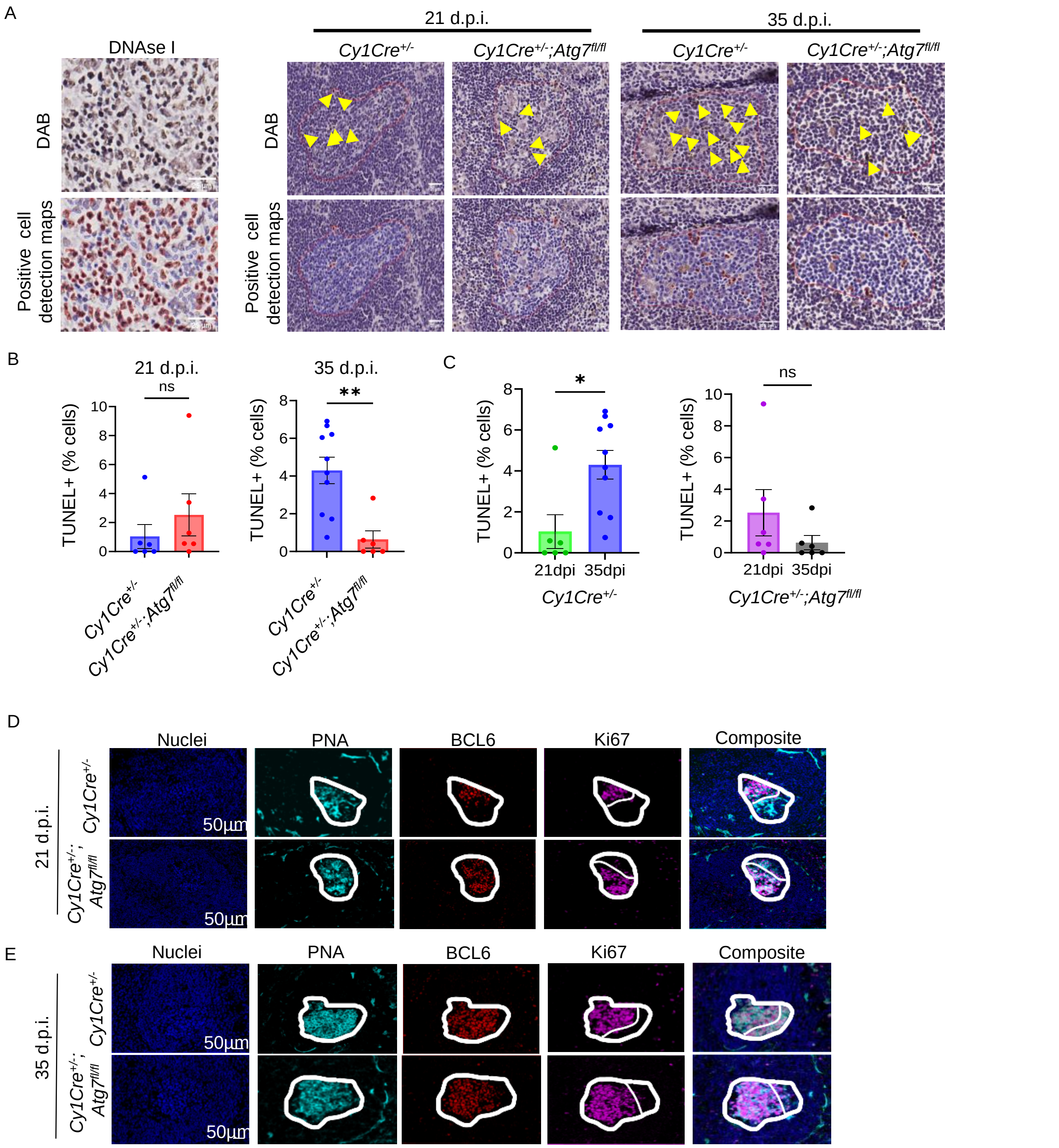

Supplemental Figure 10. Autophagy impairment results in decreased cell death

A. Positive DNAse I-treated control samples (left) and representative TUNEL staining of *Cγ1Cre+/-* and *Cγ1Cre; Atg7fl/fl* GCs at 21 d.p.i. and 35 d.p.i. (right). Apoptotic cells were DAB stained (brown, arrow) after the TUNEL assay. For quantification, a QuPath mask was used to define the positive/negative threshold of signal positivity. B. Percentage of apoptotic cells per GC comparing *Cγ1Cre+/-* and *Cγ1Cre;Atg7fl/fl* at 21 and 35 d.p.i. At least 3 GCs per spleen section were quantified from two independent immunisations. Unpaired t-test was performed. C. Percentage of apoptotic cells in GCs from *Cγ1Cre+/-* and *Cγ1Cre;Atg7fl/fl* mice comparing the day 21 and day 35 timepoints. Unpaired t-test was performed D. Pseudo-coloured images from mP-IHC staining in A. Nuclei (Blue), PNA (Cyan), BCL6 (Red), Ki67 (Magenta) and Composite images coincide in the GC areas of *Cγ1Cre+/-* and *Cγ1Cre;Atg7fl/fl* spleens after 21 d.p.i. E. Pseudo-coloured images from mP-IHC staining in C. Nuclei (Blue), PNA (Cyan), BCL6 (Red), Ki67 (Magenta) and Composite images coincide in the GC areas of *Cγ1Cre+/-* and *Cγ1Cre;Atg7fl/fl* spleens after 35 d.p.i.

Supplemental Figure 11

A

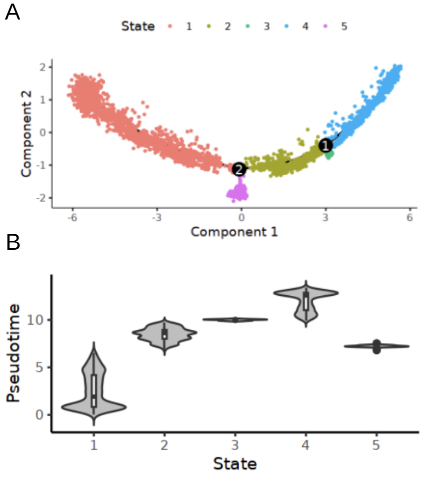

C

Cell state	1	2	3	4	5
Cγ1Cre[+/-]<br>10 d.p.i	750<br>45.42%	317<br>19.20%	123<br>7.45%	247<br>14.96%	214<br>12.96%
Cγ1Cre;Atg7[fl/fl]<br>10 d.p.i	810<br>46.73%	313<br>18.06%	98<br>5.65%	317<br>18.29%	195<br>11.25%
Cγ1Cre[+/-]<br>21 d.p.i	89<br>16.69%	37<br>6.94%	17<br>3.18%	338<br>63.41%	52<br>9.75%
Cγ1Cre;Atg7[fl/fl]<br>21 d.p.i	346<br>41.19%	65<br>7.73%	20<br>2.38%	332<br>39.52%	77<br>9.16%

D

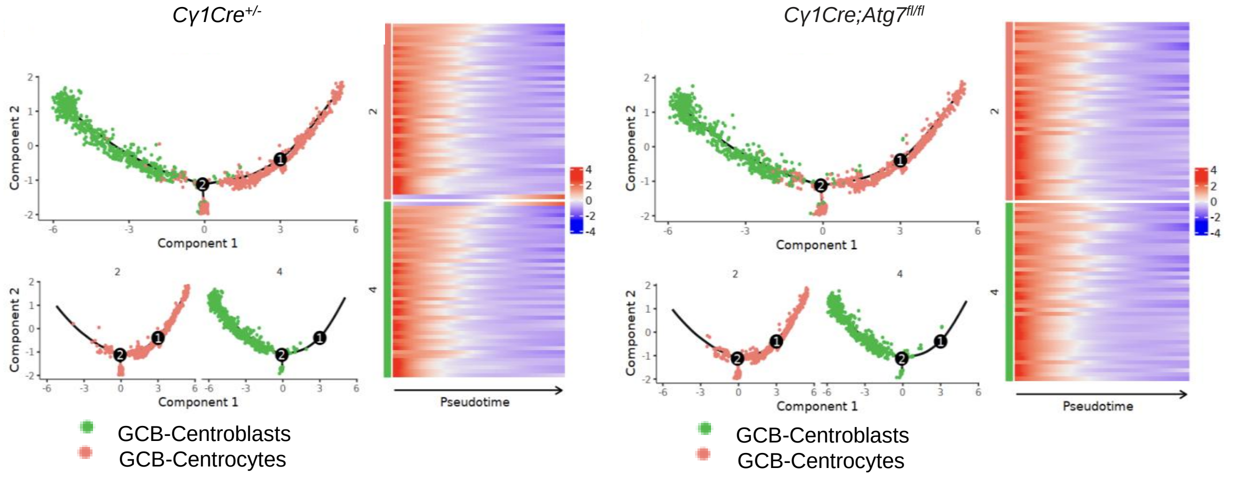

E

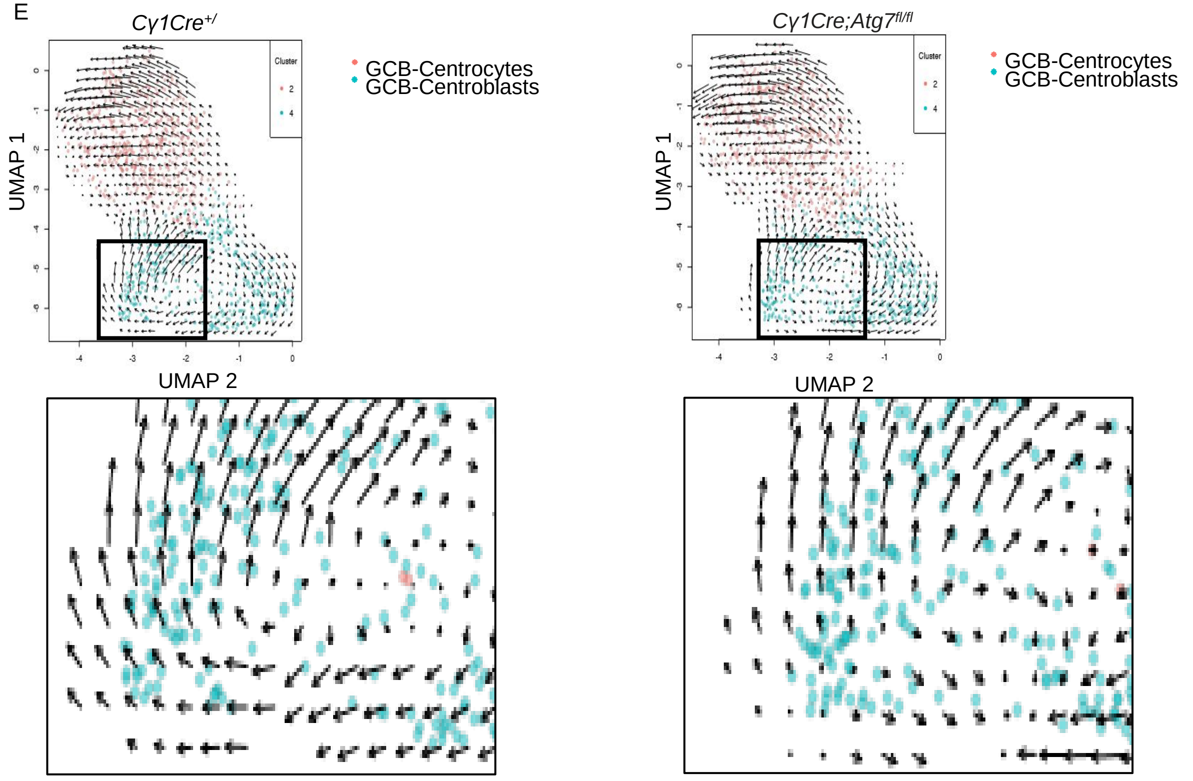

Supplemental Figure 11. Autophagy-deficient germinal centre B cells present a blockage in the centroblast to centrocyte transition.

A. Visualisation of the five distinct cell states identified along the trajectory in GCB centroblasts and GCB centrocytes. B. Violin plot showing the average and standard deviation of cells in five different states. C. Table showing the total number of cells per sample and cell state. Absolute numbers and percentages per genotype and cell state are shown in (A) and (B), respectively. D. Visualisation of 10 d.p.i *Cγ1Cre+/-* and *Cγ1Cre;Atg7fl/fl* GCB Centrobalsts (green) and GCB Centrocytes (red) single-lineage cells marked with the inferred pseudotime by Monocle. Heatmap displaying changes in gene expression across pseudotime in *Cγ1Cre+/-* and *Cγ1Cre;Atg7fl/fl.* E. RNA velocity analysis UMAP visualisation of the *Cγ1Cre+/-* and *Cγ1Cre;Atg7fl/fl* cells after 10 d.p.i.

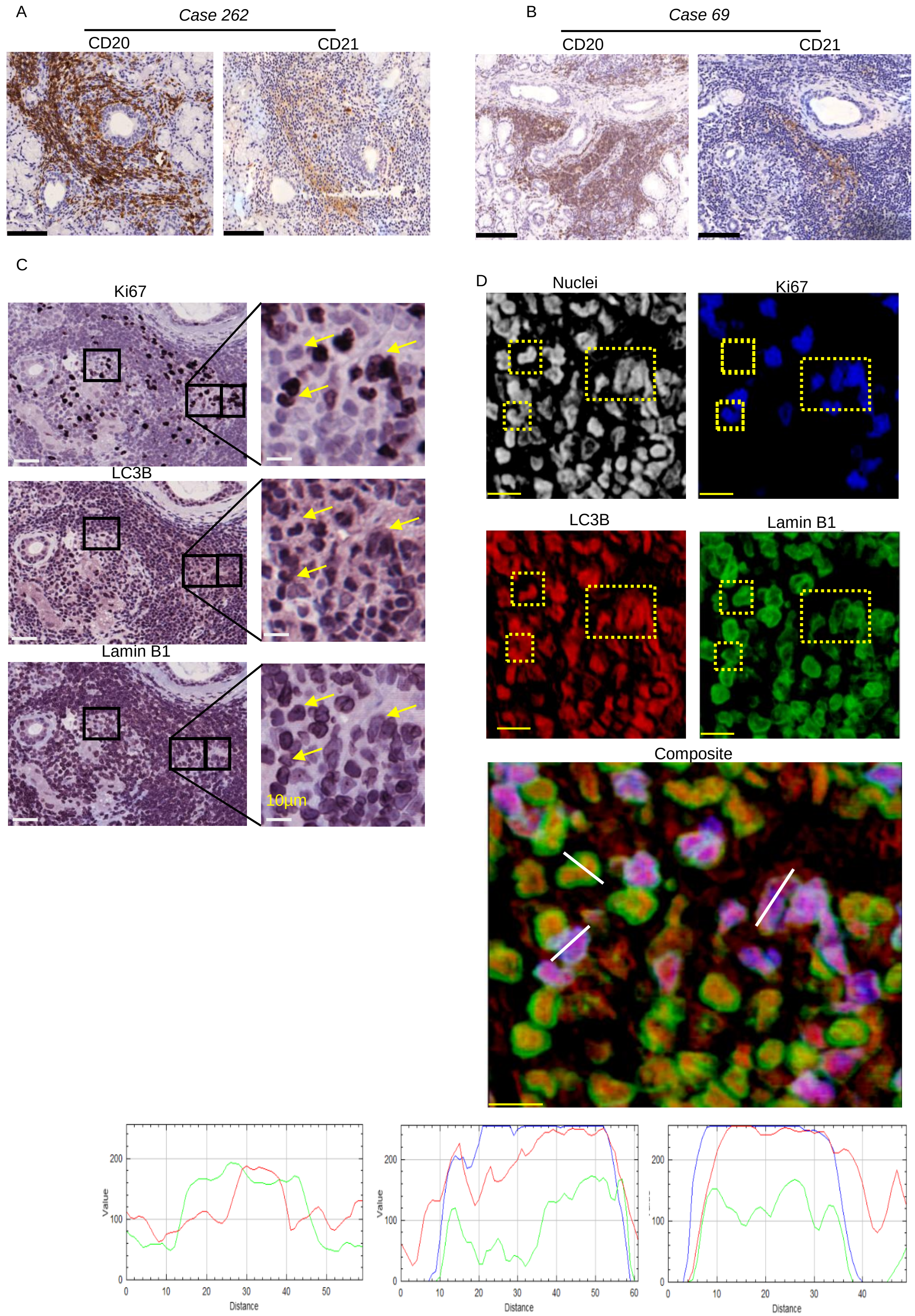
A
Case 262
CD20
CD21
B
Case 69
CD20
CD21
C
Ki67
LC3B
Lamin B1
10µm
D
Nuclei
Ki67
LC3B
Lamin B1
Composite
Value
Distance
0
100
200
10
20
30
40
50
60

Supplementary Figure 12. Sjögren's patients exhibit elevated LC3B levels alongside diminished Lamin B1 expression within ectopic germinal centres

A-B. CD20 and CD21 sequential staining in two representative SjS cases (262 and 69). Scale bar 100 µm. C. Multiplex immunohistochemistry analysis showing Ki67, LC3B and Lamin B1 in the same area in representative SjS (case 69). Scale bar 50µm. Yellow arrows indicate the same cells across the multiple stainings in the zoomed images. Scale bar 10µm. D. Multiplex immunohistochemistry showing nuclei (grey), Ki67 (blue), LC3B (red), and Lamin B1 (green) in the same representative SjS case shown in (A). Scale bar 10µm. Plot profile analysis of 3 representative cells.
