# Supplementary material for: Autophagy is an upstream mediator of chromatin dynamics in normal and autoimmune germinal center B cells
Source: J Clin Invest. 2025 May 15;135(13):e178920. doi: 10.1172/JCI178920 (PMC12208547; doi:10.1172/JCI178920)

Full unedited blot/gel for Figure 3E

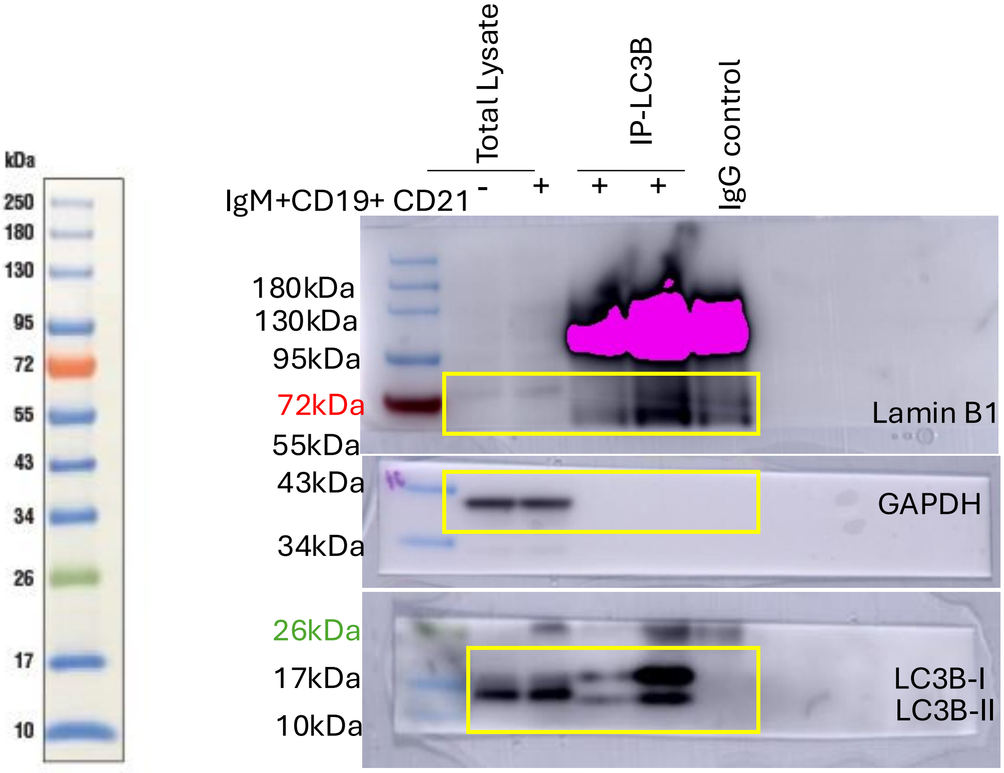

Full unedited blot/gel for Supplementary Figure 4A

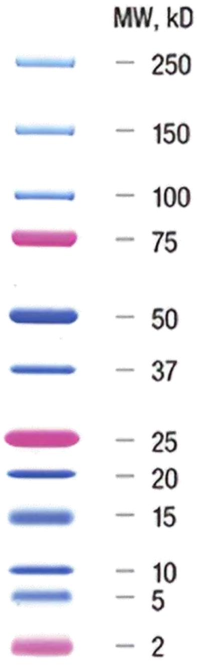

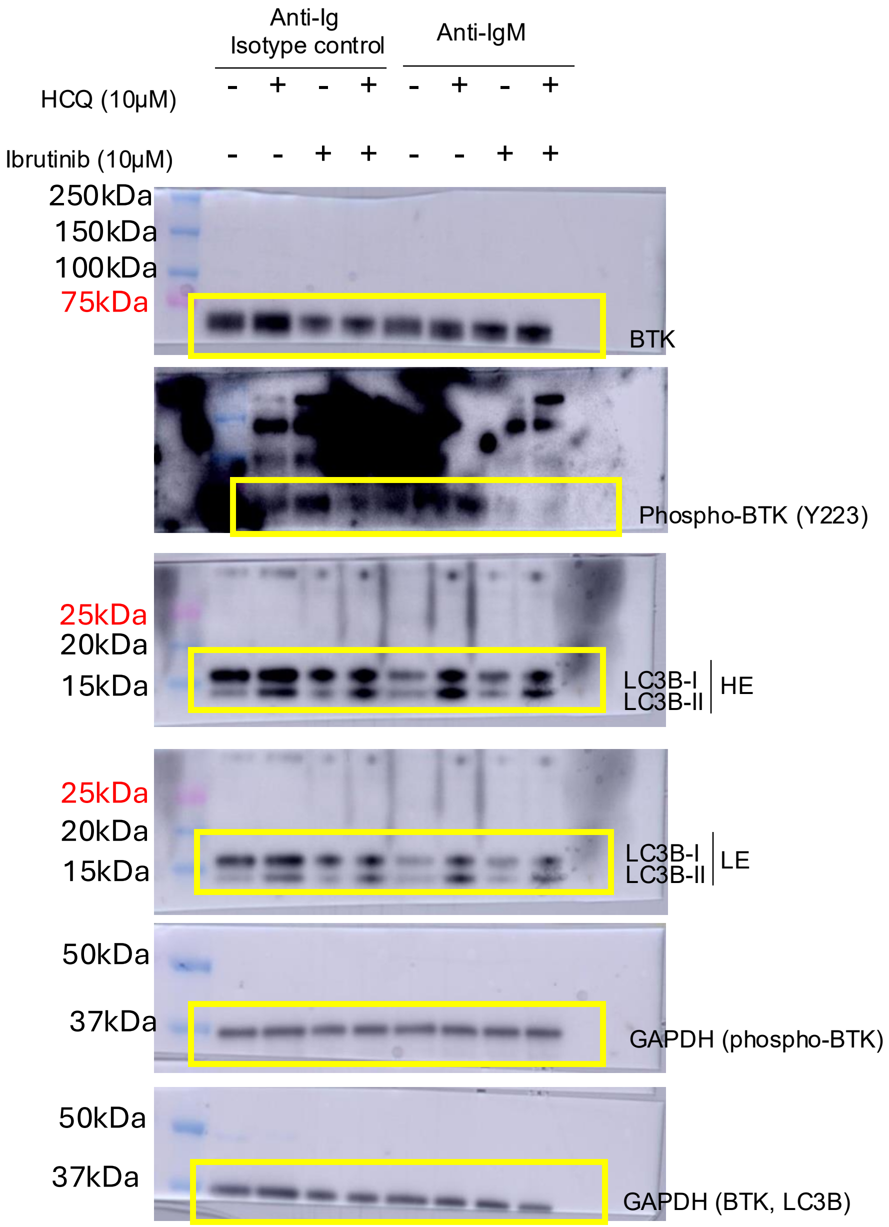

Full unedited blot/gel for Supplementary Figure 6F

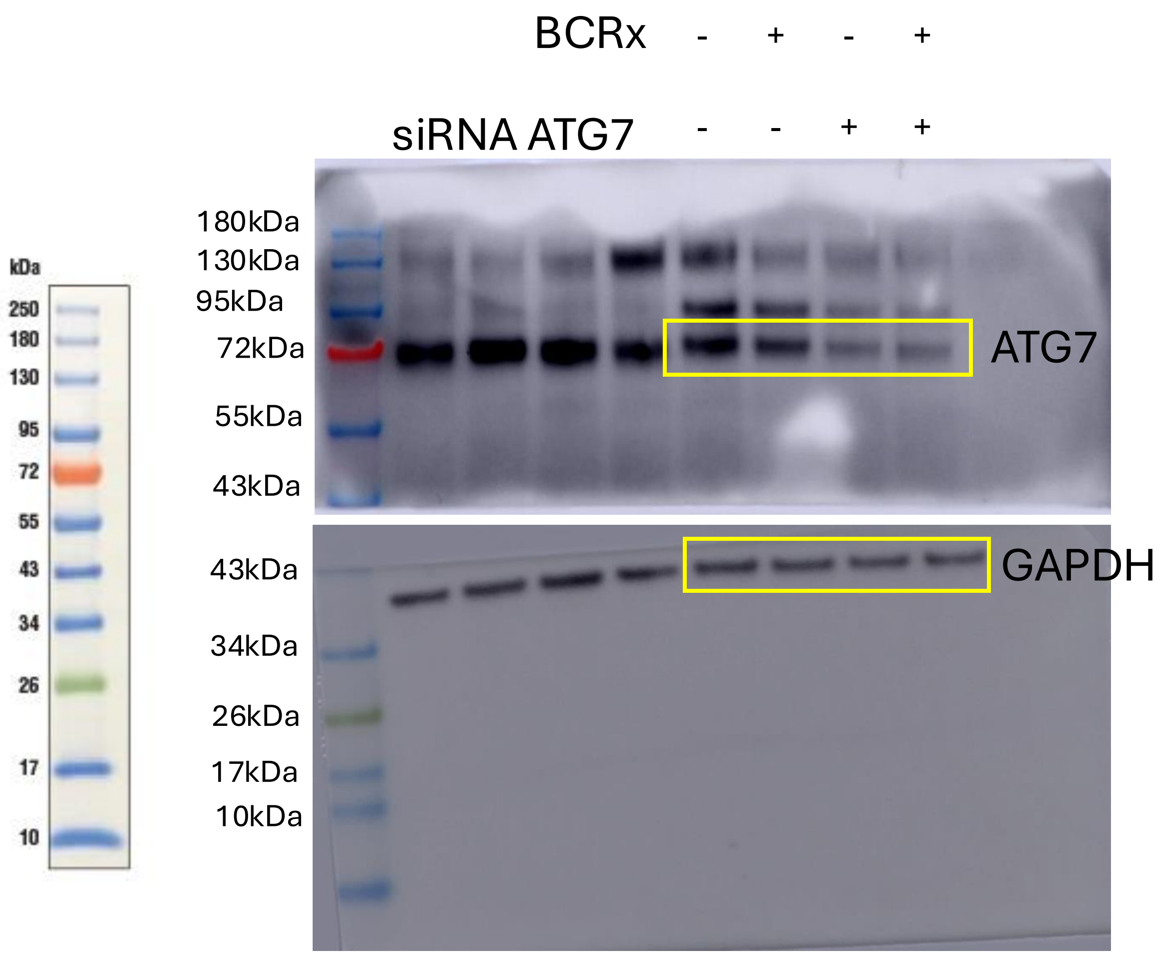

Supplement: Unedited blot and gel images [file jci-135-178920-s183.pdf]
